# Unveiling the Structural and Biochemical Characteristics of an Acidophilic β‑Mannanase from Soybean (*Glycine max*)

**DOI:** 10.1021/acs.jafc.5c03141

**Published:** 2025-09-25

**Authors:** Chun-Jung Lin, Chao-Cheng Cho, Sheng-Chia Chen, Gloria Meng-Hsuan Lin, Cheng-Yang Huang, Chun-Hua Hsu

**Affiliations:** a Department of Agricultural Chemistry, 33561 National Taiwan University, Taipei 10617, Taiwan; b Institute of Biochemical Sciences, 33561 National Taiwan University, Taipei 10617, Taiwan; c Genome and Systems Biology Degree Program, National Taiwan University and Academia Sinica, Taipei 10617, Taiwan; d Center for Computational and Systems Biology, 33561 National Taiwan University, Taipei 10617, Taiwan

**Keywords:** soybean, β-mannanase, X-ray crystallography, GmMAN19-1, branched mannans

## Abstract

Endo-1,4-β-mannanase (EC 3.2.1.78) cleaves β-1,4-mannans in cell walls, facilitating endosperm softening and seed germination. Here, we present the structural and biochemical characterization of GmMAN19-1, a GH5_7 β-mannanase from soybean (*Glycine max*), a crop with substantial agricultural importance. GmMAN19-1 is specifically expressed in cotyledons during postgermination and exhibits acidophilic activity optimal at pH 4.6 and 40 °C. Crystal structures of GmMAN19-1 were determined at 1.39 and 2.62 Å in the apo form and in complex with mannopentaose (M5), respectively. The structure adopts a canonical (α/β)_8_ TIM barrel fold with a V-shaped active site groove. Notably, two distinct M5 binding modes were identified, suggesting dual functionality involving hydrolytic and transglycosylation activities. Site-directed mutagenesis further validated key catalytic and substrate-interacting residues: E186A abolished enzymatic activity, while Q267W altered transglycosylation product profiles and enhanced activity toward branched substrates. The binding groove can accommodate galactose side chains, supporting partial activity toward galactomannans. These findings provide comprehensive insights into the substrate specificity and catalytic mechanism of plant β-mannanases and establish GmMAN19-1 as a potential candidate for applications in food processing, biomass conversion, and industrial biotechnology, particularly due to its acidophilic nature and enhanced activity toward branched mannans.

## Graphical abstract

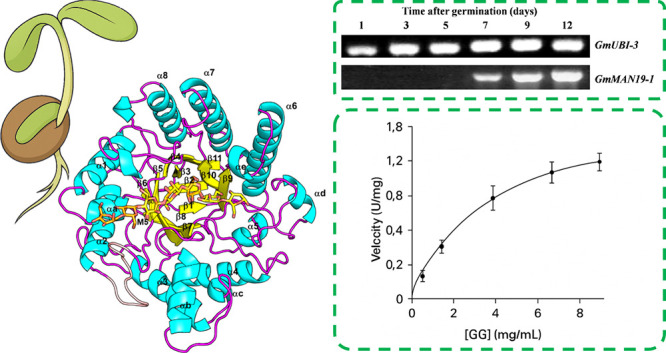

## Introduction

Soybean (*Glycine max*) is the world’s most important leguminous crop, extensively cultivated across diverse agricultural regions.^1^ Soybean meal, a byproduct of oil extraction from seeds, serves as a major protein source in livestock and poultry feeds. β-Mannan, a nonstarch polysaccharide commonly present in soybean meal-based poultry diets, has been shown to negatively impact animal growth and feed conversion efficiency. However, recent studies demonstrate that dietary supplementation with β-mannanase can alleviate the antinutritional effect of mannan in soybean meal, thereby enhancing performance in poultry and swine.^2,3^

Hemicellulose, a key structural component of plant cell walls, includes β-1,4-mannans as a major class of polysaccharides. These mannans, which include linear mannans, galactomannans, glucomannans, and galactoglucomannans, serve both as structural elements and as seed storage reserves in higher plants and algae. Endo-β-1,4-mannanases catalyze the random cleavage of internal β-1,4-mannosidic linkages in the mannan backbone, thereby releasing mannooligosaccharides. Plant-derived β-mannanase genes have been identified in various species, such as tomato (*Lycopersicon esculentum*), coffee (*Coffea arabica*), and lettuce (*Lactuca sativa*).^4–6^ Similarly, β-mannanase genes have been isolated from soybean (*Glycine max*),^7^ presenting opportunities to improve the nutritional quality of soybean-based feeds through enzymatic or genetic approaches.

Glycoside hydrolase family 5 (GH5) is one of the most functionally diverse enzyme families in the CAZy database,^8^ encompassing a wide range of substrate specificities. Based on sequence similarity, GH5 is divided into 57 subfamilies,^9^ many of which share conserved structural features and catalytic mechanisms. GH5 mannanases typically exhibit substrate preference shaped by the topology of their active sites: endoacting enzymes possess cleft-like active sites suitable for internal bond cleavage, while exoacting counterparts have pocket-shaped active sites that favor terminal residues. Although microbial GH5 mannanases from bacterial and fungal sources have been extensively characterized,^10,11^ structural information on plant-derived β-mannanases, particularly those in complex with linear mannooligosaccharides such as mannopentaose (M5) or mannohexaose (M6), remain scarce. GH5_7 (GH5 subfamily 7) denotes a group within the GH5 family primarily consisting of plant-derived endo-β-1,4-mannanases (EC 3.2.1.78), which participate in the degradation of β-mannan polysaccharides during seed germination. Some members have also been reported to exhibit transglycosylase or exo-β-mannosidase activities, reflecting functional and structural diversity within the subfamily.

In this study, we report the phylogenetic, biochemical, and structural characterization of GmMAN19-1, a GH5_7 β-mannanase from soybean. High-resolution crystal structures of GmMAN19-1 in apo form and in complex with M5 reveal two distinct binding modes within the enzyme’s cleft-shaped active site, mapping substrate interactions across subsites from +2 to −5. Additionally, we investigated the enzyme’s pH and temperature optima, substrate specificity, and transglycosylation activity, providing new insights into the structure–function relationship of plant GH5 mannanases and their potential for biotechnological applications.

## Materials and Methods

### Database Search and Sequence Analysis

Protein sequences of soybean mannanases were retrieved from Phytozome (https://phytozome-next.jgi.doe.gov/) and used as queries to perform BLASTp searches within the *Glycine max* genome (Wm82.a2.v1).^12^ For phylogenetic reconstruction, multiple sequence alignment of selected endo-β-mannanase proteins was conducted using Phylogeny.fr web server (http://www.phylogeny.fr/).^13^

Total RNA was extracted from germinating seeds (black soybean Tainan 3), root, stem, and leave using TRIzol (Sigma-Aldrich, St. Louis, MO, USA).^14^ DNA contamination was removed through DNase treatment. First-strand cDNA synthesis was performed using the ProtoScript II First Strand cDNA Synthesis Kit (New England BioLabs, NJ, US), as previously described.^15^ Using the first-strand cDNA as a template, polymerase chain reactions (PCR) were conducted with gene-specific primers to analyze the expression of GmMAN19-1 and GmMAN11.

### Protein Production and Purification

To express the mature form of GmMAN19-1, the signal peptide was predicted using SignalP,^16^ indicating that the N-terminal residues 1–27 constitute a signal peptide, with the mature protein starting at the 28th amino acid (Cysteine). The corresponding coding region was amplified by PCR and cloned into the pGEX-4T-1 vector using *Eco*RI and XhoI restriction sites to generate an N-terminal GST fusion construct. The following primers were used for PCR amplification: the forward primer (*Eco*RI site underlined) 5′-AATTGAATTCTGCATGTGAAGTGTGGGGA-3′ and the reverse primer (XhoI site underlined) 5′-AATTCTCGAGTTATGCTTAGCAATAGGATGAAGT-3′. Among the tested constructs, the GST-tagged fusion protein showed significantly higher expression levels compared to the His-tagged version. Therefore, the GST-fusion construct was chosen for large-scale expression. The recombinant plasmid was transformed into *E. coli* BL21­(DE3) cells. Cultures were grown at 37 °C with shaking until the optical density at 600 nm (OD_6__0__0_) reached approximately 0.6. Protein expression was induced with 0.5 mM isopropyl β-D-1-thiogalactopyranoside (IPTG) at 30 °C for 16 h. Cells were harvested by centrifugation at 6000 rpm for 5 min at 4 °C.

The cell pellet was resuspended in 60 mL phosphate-buffered saline (PBS), and cell disruption was performed using ultrasonic processor (ChromTech) at 30% amplitude, with 2 s on/3 s off pulse cycle, for a total of 40 min. The sample was kept on ice throughout the process to minimize thermal denaturation. The lysate was centrifuged at 14,000 rpm for 30 min at 4 °C. 1 mL of glutathione agarose resin (UBPBio, Dallas, TX, USA) was added to each 60 mL of cleared lysate, and the mixture was incubated on ice for at least 1 h with gentle mixing to ensure efficient binding of the GST-tagged GmMAN19-1.

After incubation, the resin was packed into a column. The lysate was loaded, followed by washing with 30 mL PBS to remove nonspecifically bound proteins. The GST-fusion protein was eluted using 50 mM Tris-HCl (pH 8.0) containing 10 mM reduced glutathione. To remove the GST tag, thrombin was added to the eluted fraction and incubated at 10 °C for 16 h. The digested protein sample was subsequently subjected to gel filtration chromatography using a Superdex 75 column equilibrated in PBS to separate cleaved GmMAN19-1 from the GST tag. Elution fractions were monitored at 280 nm, and protein purity was confirmed by 12% SDS-PAGE. The concentration of purified GmMAN19-1 was determined by UV absorbance at 280 nm using the calculated extinction coefficient.^17^

### Circular dichroism (CD) spectroscopy

CD measurements were performed using a Chirascan-plus qCD spectrometer (Applied Photophysics, UK). Protein samples were prepared in 20 mM sodium phosphate buffer at varying pH values (5.5–9.0). Far-UV CD spectra (190–260 nm) were recorded at 25 °C using a 20 μM protein sample in a 1 mm path-length cuvette. Each spectrum was averaged from three scans, collected at a scan rate of 20 nm/min with a 1 nm bandwidth, after subtracting the blank solvent signals.

### β-Mannanase Activity Assay

The enzymatic activity of GmMAN19-1 was assessed using the 3,5-dinitrosalicylic acid (DNS) method,^18^ which quantifies reducing sugars released from the hydrolysis of mannans. One unit (U) of β-mannanase activity was defined as the amount of enzyme required to release 1 μmol of mannose per minute. The DNS reagent consisted of 1% (w/v) DNS, 40% (w/v) potassium–sodium tartrate tetrahydrate, 10% (w/v) NaOH, and 0.05% (w/v) sodium sulfite.

Substrate solutions were prepared by dissolving 10 mg/mL of locust bean gum (LBG; Sigma-Aldrich, St. Louis, MO, USA), guar gum (GG; Sigma-Aldrich, St. Louis, MO, USA) or ivory nut mannan (INM; Megazyme, Bray, Ireland). For INM, which is poorly soluble in water, a 1% NaOH solution was used with sonication (50% amplitude, 8s on/4s off, 20 min on ice), followed by centrifugation to remove insoluble materials. For each assay, 10 μL of GmMAN19-1 enzyme solution (1 mg/mL) was mixed with 240 μL of substrate solution and incubated under the desired conditions. The reaction was terminated by adding 500 μL of DNS reagent, boiled for 10 min, and cooled to room temperature. Absorbance was measured at 540 nm using a SpectraMax iD5 plate reader (Molecular Devices, San Jose, CA, USA), and the concentration of reducing sugar was calculated based on a mannose standard curve. All assays were performed in triplicate.

To determine the optimal pH, reactions were conducted using LBG as substrate in three buffer systems: McIlvaine buffer (pH 2.6–7.0), phosphate buffer (pH 7.0–8.0), and glycine-NaOH buffer (pH 8.6–10.6). For temperature profiling, the substrate solution was pre-equilibrated to target temperatures (25–50 °C), and reactions were carried out at each temperature for 10 min. The pH and thermal stability were evaluated by preincubating the enzyme in buffers or at various temperatures, respectively, and measuring residual activity. Each experiment was conducted in triplicate.

### Enzyme Kinetics Analysis

To determine steady-state kinetic parameters, enzyme activity was measured using the DNS assay^18^ under optimized conditions (pH 4.6, 40 °C unless otherwise specified). The enzyme reaction mixture contained 10 μL of GmMAN19-1 (1 mg/mL stock), resulting in a final enzyme concentration of approximately 0.04 mg/mL in a total volume of 250 μL. Time-course assays confirmed that linear reaction conditions were achieved within 10 min at 40 °C and 15 min at 30 °C for LBG, and within 20 min (40 °C, 10 μg enzyme) or 10 min (30 °C, 20 μg enzyme) for GG. Kinetic measurements were thus conducted using 5- or 10 min reaction times under steady-state conditions.

Substrate concentrations ranged from 1 to 10 mg/mL. Michaelis–Menten kinetic parameters (K_M_, *V*_max_) were determined for LBG, GG and INM at 30 and 40 °C using nonlinear regression fitting in Prism 8.0. All kinetic experiments were performed in triplicate.

### Site-Directed Mutagenesis

Site-directed mutants of GmMAN19-1 (E186A, Q267W) were generated using overlap-extension PCR with the following primers: for the E186A mutation, E186A-F (5′-GAACTTATGAATGCGCCCCGTTCCCAA-3′) and E186A-R (5′-TTGGGAACGGGGCGCATTCATAAGTTC-3′); for the Q267W mutation, Q267W–F (5′-ATCTCTACCCTGAATGGTGGTTGCCAGGCTC-3′) and Q267W-R (5′-GAGCCTGGCAACCACCATTCAGGGTAGAGAT-3′). The mutations were confirmed by sequencing. Recombinant proteins were expressed in *E. coli* BL21­(DE3) and purified following the same protocol used for the wild-type (WT) protein.

### Thin Layer Chromatography (TLC)

Transglycosylation activity was analyzed using thin layer chromatography (TLC) following previously described methods,^19,20^ with minor modifications. Reactions were performed using 1 μM enzyme and 25 mM mannopentaose (M5; Megazyme) under optimal assay conditions. Samples were collected at designated time points (5 min to 4 h) to monitor the progression of product profiles. Each sample was diluted 15-fold with distilled water and heat-inactivated in a boiling water bath for 5 min prior to spotting.

For TLC analysis, 20 μL of each sample was applied onto silica gel 60G F254 plates (20 × 20 cm, Merck). To improve detection sensitivity, 2 μL aliquots of the reaction mixture were sequentially spotted onto the same location of a TLC plate, allowing each to air-dry before the next was applied. This stepwise layering accumulated a total of 20 μL without excessive spreading. The mobile phase consisted of n-butanol, acetic acid, and water (2:1:1, v/v/v), pre-equilibrated in the developing chamber for 30 min. After development, the plates were air-dried and visualized by spraying with a 95:5 (v/v) methanol–sulfuric acid solution (Sigma-Aldrich, St. Louis, MO, USA). Plates were then heated at 120 °C for 5 min, allowing the appearance of brown to black spots corresponding to saccharide species.

### High-Performance Anion Exchange Chromatography (HPAEC)-Pulsed Amperometric Detector (PAD)

To confirm the hydrolytic capability and transglycosylation of GmMAN19-1, enzyme-treated product from M5 were analyzed using HPAEC-PAD (high-performance anion-exchange chromatography with pulsed amperometric detection) Commercial M1 and M5 standards were used as references for retention time and quantification. Analyses were performed on a DIONEX ICS-5000 system (Thermo Fisher Scientific) equipped with a pulsed amperometric detector. Separation was achieved using a CarboPac PA200 analytical column (3 × 250 mm) maintained at 30 °C. Samples (10 μL each) were injected and eluted with 45 mM NaOH at an isocratic flow rate of 0.5 mL/min for 15 min.

### Mass Spectrometry

To detect possible transglycosylation products, the reaction mixtures were analyzed using ESI and MALDI mass spectrometry. For ESI analysis, a 10 μL aliquot of the reaction mixture was directly infused into a Waters SYNAPT HDMS Q-TOF mass spectrometer (Waters Corp., Manchester, UK) via the *in situ* nanospray interface. Spectra were acquired in the positive ion mode with a spray voltage of 2 kV.

For MALDI, 5 μL of each sample was mixed with 5 μL of a matrix solution containing 10 mg/mL 2,5-dihydroxybenzoic acid (DHB) in 100% (v/v) methanol. Two μL of the resulting mixture were spotted onto a standard steel MALDI plate and air-dried. Mass spectra were acquired using a Bruker ultrafleXtreme MALDI-TOF mass spectrometer (Bruker Daltonics, Bremen, Germany) equipped with an Nd:YAG laser (255 nm, 200 Hz repetition rate). Data were collected in the positive reflector mode with an accelerating voltage of 20 kV and grid voltage of 16 kV.

### Crystallization and Data Collection

Crystallization trials for GmMAN19-1 were conducted at 283 K using the sitting-drop vapor diffusion method. Commercial crystallization screen kits and Cryschem plate (HR3-160, HAMPTON) were used for screening. Each crystallization drop consisted of 1 μL of GmMAN19-1 (17 mg/mL) mixed with an equal volume of reservoir solution and equilibrated against 300 μL of the same solution. Crystals of apo GmMAN19-1 were obtained in a solution containing 0.2 M Ammonium acetate, 0.1 M Sodium acetate trihydrate pH 4.6 and 30% PEG 4000 over a period of 5 days at 283 K.

To obtain a substrate-bound complex, a soaking experiment was performed using mannopentaose (M5) to investigate key amino acid residues involved in substrate binding and catalysis. M5 was prepared as a 200 mM stock solution in ddH_2_O and added to the crystallization drop containing GmMAN19-1 crystals to achieve a final concentration of 100 mM. The crystals were soaked for 5 min, ensuring that the substrate did not disrupt crystal integrity.

For diffraction experiments, crystals were cryoprotected in reservoir solution supplemented with 20% glycerol and flash-cooled in liquid nitrogen. X-ray diffraction data for the apo and M5-bound forms of GmMAN19-1 were collected at 100 K using an ADSC Quantum-315r CCD area detector on the BL13B1 beamline and an ADSC Quantum-210 CCD area detector on the BL13C1 beamline (NSRRC, Taiwan), respectively. For the apo form, each frame was recorded with a 0.5° oscillation angle over a 2 s exposure time at a crystal-to-detector distance of 92 mm. The crystal belonged to the primitive hexagonal space group P6_3_, with unit cell parameters *a* = 83.442 Å, *b* = 83.442 Å, *c* = 87.182 Å; α = 90°, β = 90°, and γ = 120°. For the M5-bound form, each frame was recorded with a 1° oscillation angle over a 40 s exposure time at a crystal-to-detector distance of 400 mm. The crystal belonged to the same space group, with unit cell parameters *a* = 83.742 Å, *b* = 83.742 Å, *c* = 86.105 Å; α = 90°, β = 90°, and γ = 120°. Diffraction data were processed using HKL2000.^21^

### Structure Determination and Refinement

The structures of apo GmMAN19-1 and its M5 complex were determined by molecular replacement using Phaser^22^ within the PHENIX suite,^23^ employing the LeMAN4 protein structure (PDB ID: 1RH9)^24^ as the search model. Initial models were manually adjusted in COOT^25^ by inspecting σ-weighted electron density maps (2mFo-DFc and mFo-DFc) and further refined with phenix.refine^26^ until *R*_work_ and *R*_free_ values converged. Structural validation was conducted using PROCHECK.^27^ The active site volume was calculated with CAVER,^28^ and molecular visualizations were generated using PyMOL (version 1.7, Schrödinger, LLC). Protein–ligand interactions were analyzed with LigPlot.^29^ Data collection and refinement statistics are summarized in Table 1.

**Table 1:** X-ray Data Collection and Refinement Statistics for GmMAN19-1 in the Apo Form and in Complex with M5

	GmMAN19-1	GmMAN19-1/M5
data collection	PDB: 9M20	PDB: 9M21
wavelength (Å)	1.0903	0.9762
resolution range	23.94–1.39 (1.44–1.39)	26.12–2.62 (2.72–2.62)
space group	P6_3_	P6_3_
unit cell parameters *a, b, c* (Å)	84.442, 84.442, 87.182,	83.742, 83.742, 86.105
α, β, γ (°)	90, 90, 120	90, 90, 120
total reflections	441419	35230
unique reflections	70703	10181
multiplicity	6.2 (6.0)	3.5 (1.8)
completeness (%)	99.7 (99.9)	98.6 (90.4)
*R*_merge_ ^a^(%)	6.0 (49.1)	18.8 (37.7)
*I*/σ	28.56 (3.89)	8.15 (2.57)
refinement statistics		
*R*_work_	0.127 (0.239)	0.157(0.214)
*R*_free_	0.159 (0.236)	0.240 (0.335)
macromolecules	2964	2964
ligands	8	112
solvent	632	115
protein residues	371	371
R.m.s.d., bonds length (Å)	0.005	0.010
R.m.s.d., angles	0.87	1.04
average B-factor (Å^2^)	16.76	31.49
Ramachandran plot		
most favored (%)	97.83	95.66
allowed (%)	2.17	4.07
Outlier (%)	0.00	0.27^b^

a *R*_merge_ = Σ*h*Σ*i*|*Ih*,*i* – *Ih*|/Σ*h*Σ*iIh*,*i*, where *Ih* is the mean intensity of the *i* observations of symmetry related reflections of *h*.

b The residue of GmMAN19-1/M5 in outlier of Ramachandran plot is Proline 84.

## Results

### Identification and Phylogenetic Analysis of GmMAN19-1

β-Mannanases are essential enzymes involved in the hydrolysis of mannans, a major class of seed storage polysaccharides. To identify potential β-mannanase genes in soybean (*Glycine max*), we performed genome-wide sequence mining using the tomato (*Lycopersicon esculentum*) β-mannanase LeMAN4 as a query. A total of 21 putative β-mannanases were identified in the soybean genome (Table S1), consistent with previous reports,^30^ who characterized one member for soybean meal improvement. Phylogenetic analysis revealed that these genes cluster within the broader clade of plant β-mannanases and group with functionally characterized homologues from *Arabidopsis thaliana* and *L. esculentum* (Figure S1A).

Among the identified candidates, GmMAN19-1 was selected for further functional characterization based on its high expression level and putative involvement in seed germination. Tissue-specific RT-PCR analysis demonstrated that GmMAN19-1 is exclusively expressed in cotyledons, with no detectable expression in roots, stems, or leaves (Figure S1B). A temporal expression profile during seed germination revealed that GmMAN19-1 is first expressed at 7 days postgermination and continues to increase as germination progresses (Figure S1C), implicating its role in mobilizing carbohydrate reserves during early seedling development.

To assess potential functional divergence, we also analyzed the expression of another soybean β-mannanase gene, *GmMAN11*. Unlike *GmMAN19-1*, *GmMAN11* exhibited a distinct expression profile, suggesting specialization in different physiological processes, potentially including radicle emergence. Taken together, these data suggest that GmMAN19-1 plays a specific role in postgermination cotyledon function, likely facilitating reserve mobilization to support early seedling growth.

### Biochemical Characterization of Recombinant GmMAN19-1

Recombinant GmMAN19-1 was expressed in *E. coli* as an N-terminal GST-tagged fusion protein. After affinity purification, the GST tag was removed by thrombin cleavage. The resulting protein was further purified by gel filtration chromatography to separate GmMAN19-1 from the GST tag. The elution profile revealed three distinct peaks (Figure S2A), and SDS-PAGE analysis confirmed that the second peak (P2) contained the cleaved GmMAN19-1 (Figure S2B). After protein concentration, the purified enzyme reached a final concentration of approximately 10 mg/mL. The purified protein, harboring an additional Gly-Ser dipeptide at the N-terminus, appeared as a single band of ∼ 40 kDa on SDS-PAGE (Figure S2C).

Enzymatic activity assays revealed that GmMAN19-1 exhibits optimal catalytic activity at pH 4.6, retaining over 60% activity across the pH range of 4.0–6.0 (Figure 1A). Circular dichroism (CD) spectroscopy indicated partial or complete denaturation of the secondary structure under strongly acidic conditions, correlating with activity loss (Figure 1B). In contrast, the enzyme maintained structural integrity and activity under neutral and mildly alkaline conditions.

**Figure 1:**
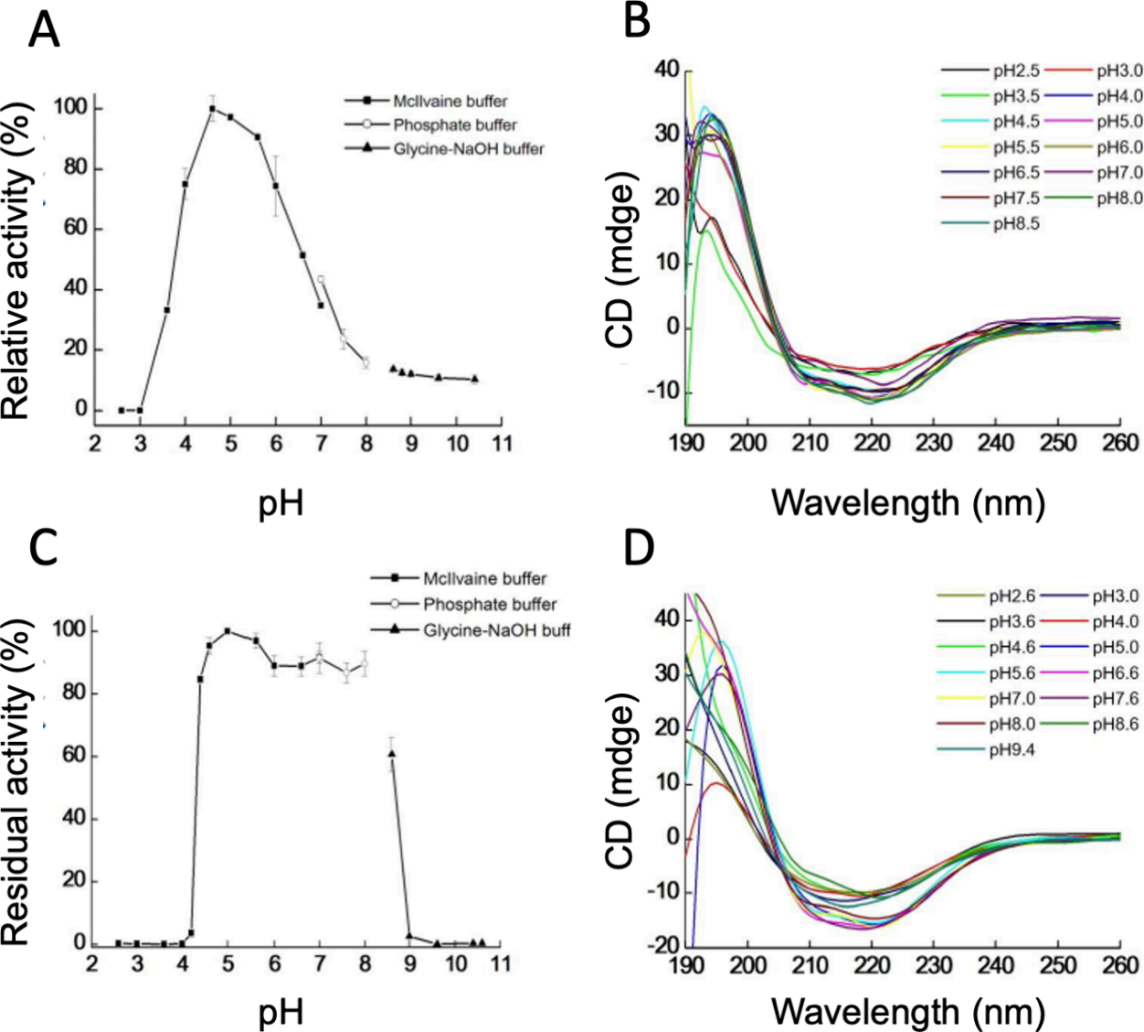
pH dependence and stability of GmMAN19-1. (A) The optimal pH for GmMAN19-1 activity was determined by measuring enzyme activity across a pH range of 2.0–10.6. (B) Circular dichroism (CD) spectra of GmMAN19-1 at different pH values. (C) pH stability of GmMAN19-1 was assessed by preincubating the enzyme in buffers of varying pH at 4 °C for 16 h, followed by activity measurement. (D) CD spectra of GmMAN19-1 after 16 h of incubation at different pH values. β-Mannanase activity was measured using the DNS method with LBG as the substrate.

To evaluate pH stability, GmMAN19-1 was preincubated at 4 °C in buffers of varying pH for 16 h. The enzyme retained over 80% of its initial activity after exposure to pH 4.6–8.0 (Figure 1C), consistent with CD spectral data showing a well-folded conformation within this range (Figure 1D).

Temperature profile analysis showed that GmMAN19-1 exhibited maximal activity at 40 °C, retaining over 90% activity between 40–45 °C for 1 h (Figure 2A,B). Activity declined to 50% after 30 min at 50 °C and was completely lost at 55 °C and above within 20 min (Figure 2B). Thermal denaturation monitored by CD at 222 nm revealed a melting temperature (Tm) of 45 °C (Figure 2C), indicating moderate thermal stability and limited heat resistance. To further understand the molecular basis underlying this activity and thermal profile, we next solved the crystal structure of GmMAN19-1.

**Figure 2:**
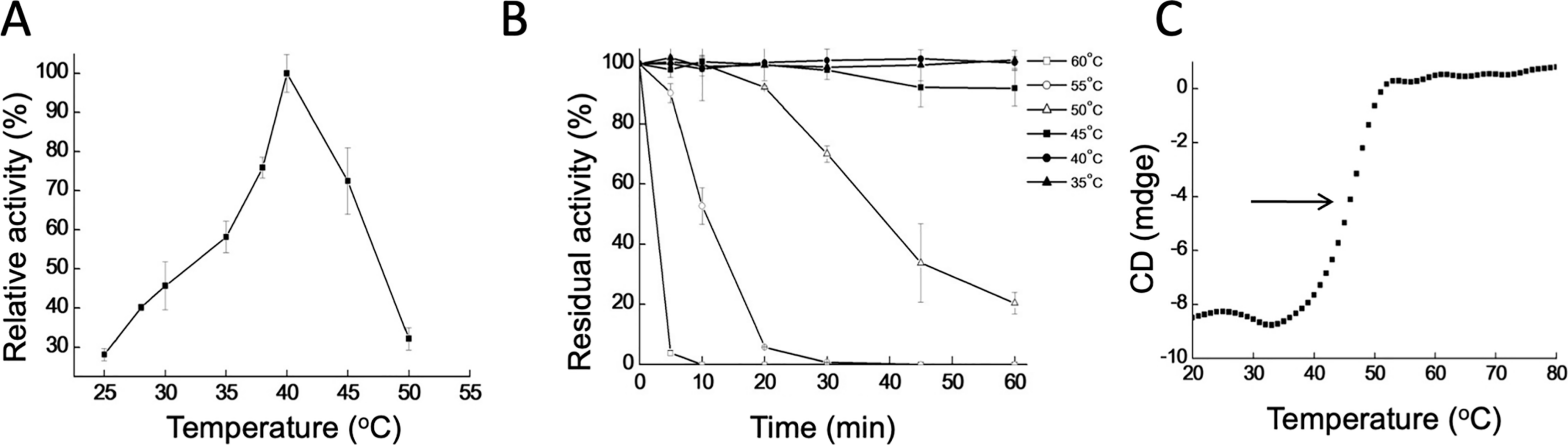
Temperature dependence and stability of GmMAN19-1. (A) The optimal temperature for GmMAN19-1 activity was determined by measuring enzyme activity at temperatures ranging from 25 to 50 °C. (B) Thermal stability of GmMAN19-1 was assessed by preincubating the enzyme at various temperatures for different durations before measuring residual activity. (C) Thermal denaturation of GmMAN19-1 was analyzed using circular dichroism (CD) spectroscopy at 222 nm in 25 mM phosphate buffer (pH 7.5). β-Mannanase activity was measured using the DNS method with LBG as the substrate.

### Dual Hydrolysis and Transglycosylation Activity of GmMAN19-1

To further elucidate the mode of action of GmMAN19-1, the enzyme’s product profile was analyzed following hydrolysis of mannopentaose (M5) under standard reaction conditions. Time-course monitoring using thin-layer chromatography (TLC) revealed the formation of oligosaccharide products (Figure S3). Notably, new saccharide bands appeared below the M5 standard within 5 min and increased in intensity over 1h, indicating the rapid generation and accumulation of elongated mannooligosaccharides via transglycosylation. The observed migration pattern was consistent with products larger than M5. Bands corresponding to mannobiose (M2), mannotriose (M3), and mannotetraose (M4) were also detected, reflecting concurrent hydrolytic activity.

To resolve and confirm the identities of these products, high-performance anion-exchange chromatography with pulsed amperometric detection (HPAEC-PAD) was performed. The M5 reaction produced both smaller and larger molecular weight peaks compared to the M5 standard (Figure S4). The appearance of later-eluting peaks supported the formation of transglycosylation products with a degree of polymerization (DP) greater than five, corroborating the TLC results.

Further structural validation was achieved via electrospray ionization (ESI)- and matrix-assisted laser desorption/ionization (MALDI)- time-of-flight (TOF) mass spectrometry. In ESI-TOF spectra (Figure S5A,B), in addition to signals for M2 (*m*/*z* 365.1), M3 (*m*/*z* 527.2), M4 (*m*/*z* 689.2), and M5 (*m*/*z* 851.2), a new peak at *m*/*z* 1013.4 corresponding to [M6 + Na]^+^ confirmed elongation beyond the original substrate. MALDI analysis further supported these findings, revealing peaks at *m*/*z* 851.3, 1013.4, and 1175.4, corresponding to [M5 + Na]^+^, [M6 + Na]^+^, and [M7 + Na]^+^, respectively (Figure S5C,D), confirming the generation of linear manno-oligosaccharides with DP up to seven. Collectively, these results demonstrate that GmMAN19-1 possesses bifunctional activity, catalyzing both hydrolysis and transglycosylation in the presence of an appropriate oligosaccharide donor.

During time-course analysis of M5 hydrolysis (Figure S3), we observed the formation of M4 but not M1 (Mannose) within the initial 4 h. This pattern suggests that GmMAN19-1 preferentially performs internal cleavage to release M4, while the terminal M1 unit may be slowly released, further degraded, or remain below the detection threshold. This observation highlights the enzyme’s catalytic bias toward generating longer oligosaccharide products.

### Overall Structure of GmMAN19-1

Following functional validation of heterologously expressed GmMAN19-1 through kinetic analyses, we sought to determine its three-dimensional structure to gain insights into substrate specificity and catalytic mechanism. Given the limited structural data available for plant-derived β-mannanases, structural elucidation of GmMAN19-1 holds particular significance. Crystals of purified GmMAN19-1 were successfully obtained through screening and optimization of crystallization conditions (Figure S6).

The crystal structure of GmMAN19-1 was solved in its apo form at 1.39 Å resolution. The enzyme adopts a canonical (α/β)_8_-TIM barrel fold, characteristic of glycoside hydrolase family 5 (GH5) (Figure 3A). The polypeptide chain of GmMAN19-1 consists of 13 α-helices and 11 β-strands (Figure 3B). The active site forms a V-shaped groove optimized for mannan substrate binding. The catalytic acid/base and nucleophile residues, E186 and E304, are positioned on β9 and β12, respectively, at the center of the catalytic groove. Overall, the conserved GH5 scaffold and the strategic positioning of catalytic residues in GmMAN19-1 underscore its functional adaptation for mannan recognition and cleavage, establishing a robust framework for subsequent substrate-bound structural analyses.

**Figure 3:**
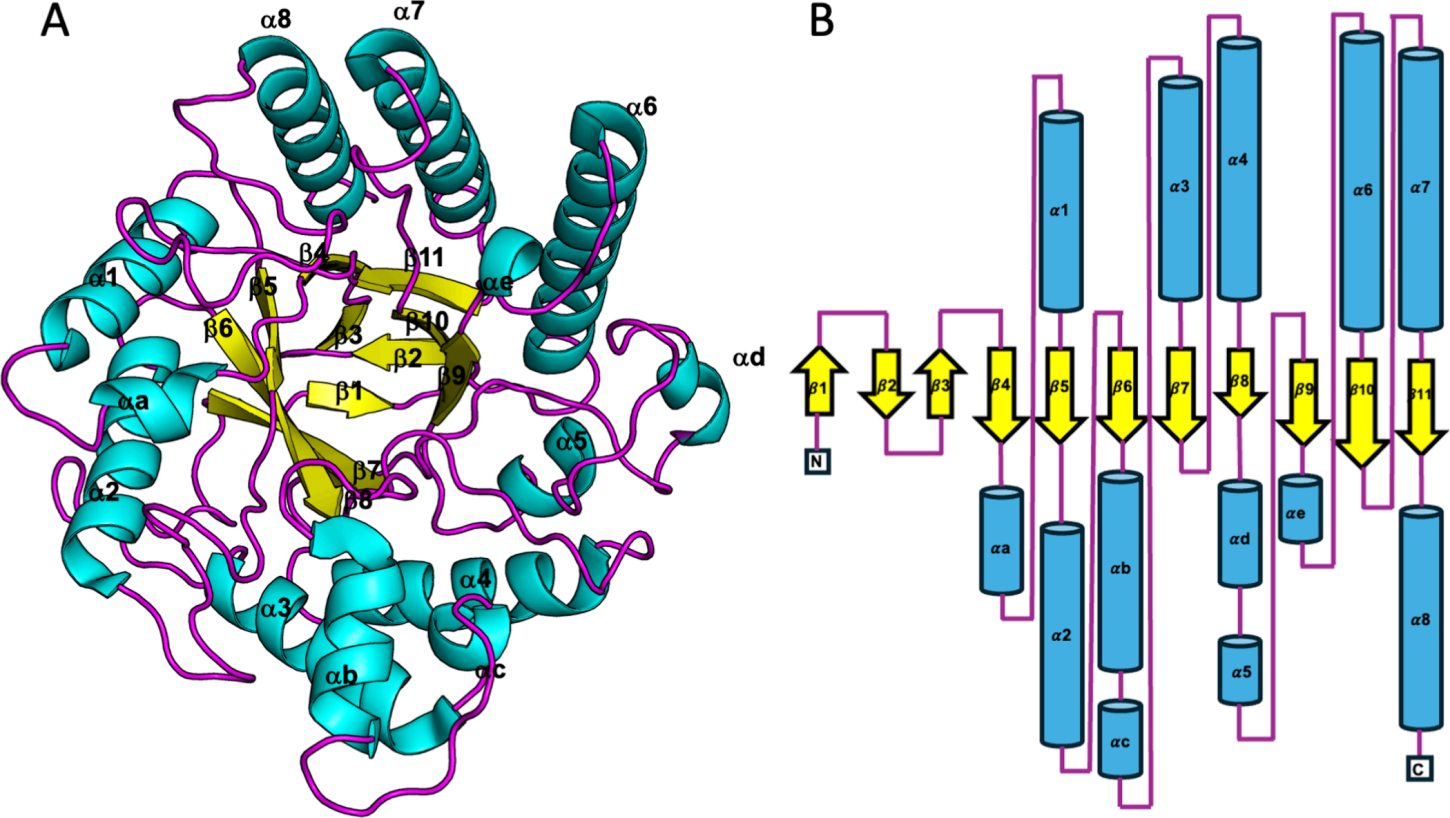
Overall structure of GmMAN19-1. (A) Cartoon representation of the GmMAN19-1 overall structure with α-helix, β-strand, and loop colored as cyan, yellow, and purple, respectively. (B) Topology diagrams use the same color scheme as the cartoon representations.

### Mannooligosaccharides Binding and Substrate Recognition Mechanism

To explore substrate recognition, crystals of GmMAN19-1 were soaked with linear mannooligosaccharides (M5) at 0.1 M for 5 min. Phase determination using the apo-form GmMAN19-1 structure revealed strong electron density corresponding to mannose residues extending along the V-shaped substrate-binding groove. Given the general catalytic mechanism, ligand soaking may lead to a covalent intermediate resulting from nucleophilic attack. To validate the structural interpretation, the absence of electron density connectivity between the nucleophile residue E304 and M5 molecule (Figure S7A) confirms that the complex represents the enzyme–substrate/product binding state rather than a covalent intermediate.^24,31^ Further analysis of the electron density revealed seven hexose rings along the active site groove (Figure S7B,C). A cartoon representation of the GmMAN19-1/M5 complex provided a clearer visualization of the substrate-binding position (Figure 4A), while a surface representation highlighted key amino acid residues involved in M5 binding (Figure 4B).

**Figure 4:**
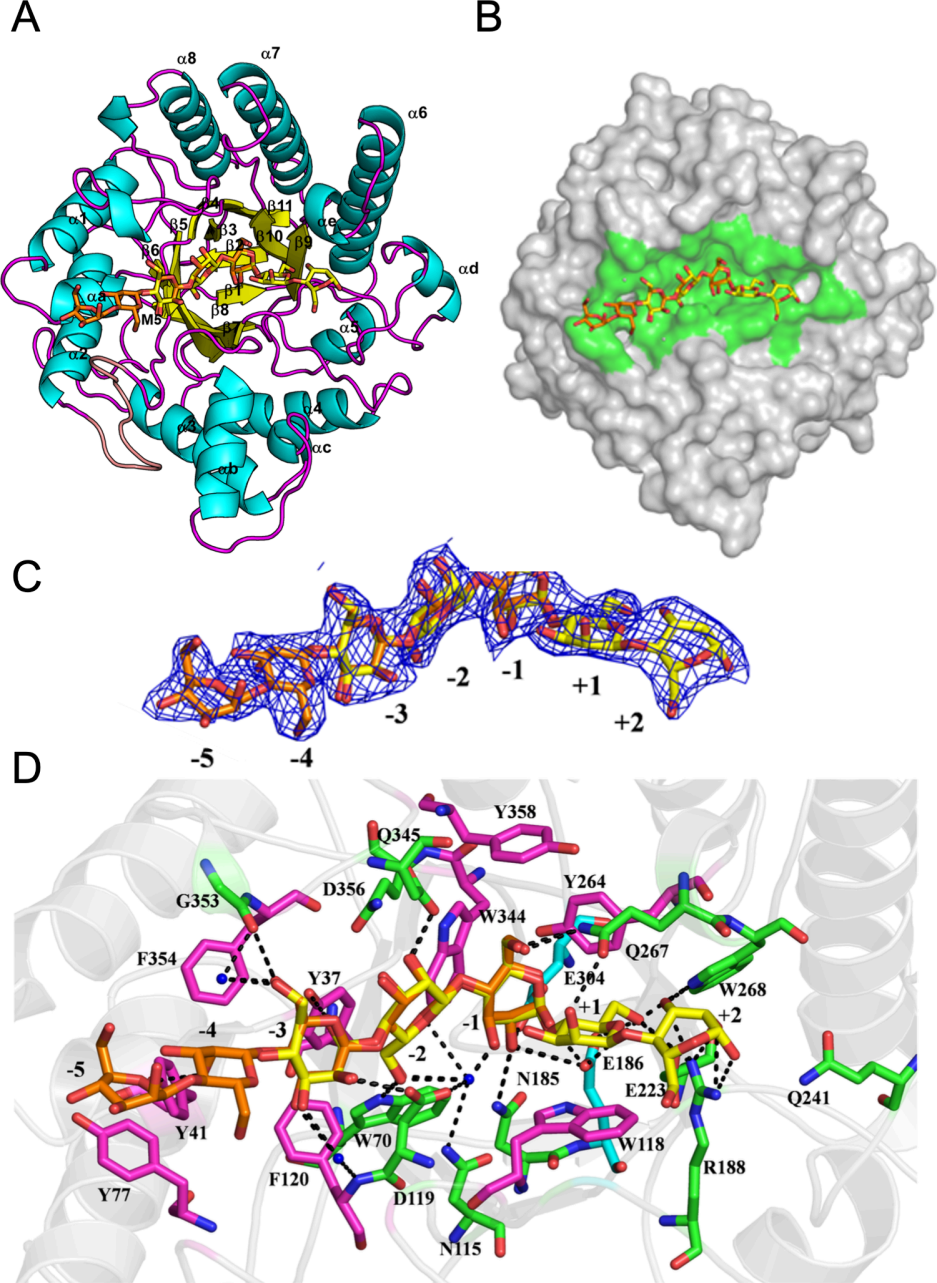
Structure of GmMAN19-1 in complex with mannopentaose (M5). (A) The GmMAN19-1/M5 complex structure reveals two M5 molecules bound within the active-site region. Mannopentaose is depicted as sticks, with carbon atoms in yellow, and the 2Fo-Fc electron density map is contoured at 1.0 σ. (B) Surface representation of GmMAN19-1, highlighting the mannopentaose (yellow carbons) positioned within the active-site cleft (green surface). (C) Close-up view of the bound mannopentaose, illustrating two distinct binding modes. The 2Fo-Fc electron density map is contoured at 1.0 σ. (D) Interactions of the substrate in the active site of GmMAN19-1/M5. Green residues form hydrogen bonds with mannopentaose, whereas magenta residues interact with the ligand via hydrophobic and van der Waals forces. Cyan residues represent catalytic residues (E186 and E304). Mannopentaose molecules are shown as sticks, with carbon atoms in yellow and orange, while water molecules are depicted as blue spheres.

The mannose backbone adopted a bent configuration centered between subsites −1 and +1. The electron density map of M5 indicated two possible binding arrangements. In one configuration, M5 extended across subsites −5 to −1, while in another, it occupied subsites −3 to +2. These observations suggest that GmMAN19-1 accommodates substrates across subsites −5 to +2. The M5 backbone adopts a V-shaped configuration, with subsite −1 serving as the pivot point (Figure 4C). Structural superposition of the apo and M5-bound GmMAN19-1 structures showed no significant conformational changes upon substrate binding, suggesting a preorganized active site optimized for substrate recognition and catalysis.

The interaction between GmMAN19-1 and M5 was analyzed using LigPlot program,^29^ which provided detailed binding site information (Figure S8). To further explore key binding-site residues, we examined their interactions at each subsite. At the −1 subsite, N185 forms a hydrogen bond with the C2-OH group of mannose, which helps stabilize the axial orientation unique to mannose. This interaction, along with hydrophobic contacts from Y264, W344, and Y358, defines the core catalytic binding. At the +1 subsite, W118 provides stacking stabilization, while Q267 and E223 form hydrogen bonds with the C2-OH and C6-OH of the adjacent mannose residue, respectively. The +2 subsite is stabilized by a hydrogen bond triad involving R188 and W268, reinforcing binding at the nonreducing end.

Notably, the mannose residue at subsite −1 adopts a skew-boat conformation, whereas the other mannose residues exhibit a standard chair conformation. This skew-boat conformation positions the 2-OH group in a pseudoequatorial geometry, optimal for nucleophilic attack by E304, and consistent with formation of a covalent glycosyl-enzyme intermediate typical for retaining mechanisms

In the substrate-binding subsites (−3 to −5), Y77, F120, and F354 contribute hydrophobic stacking interactions, with their aromatic rings nearly parallel to the sugar rings. Additionally, Y41 (OH) and D119 (Oδ1) form hydrogen bonds with the C2-OH of the −3 subsite sugar, while Y37 (OH) interacts with the C5-OH group. At the −2 subsite, D356 (Oδ1) hydrogen bonds with the C2-OH group of the sugar. These findings indicate that GmMAN19-1 employs an extensive hydrogen bond network and hydrophobic interactions to stabilize substrate binding and enhance affinity. This combination ensures a stable enzyme–substrate interaction, facilitating efficient hydrolysis. Additionally, several water molecules at the substrate-binding site form hydrogen bonds with the sugar molecule (Figure 4D).

### MD Simulation Supports Dual Binding Modes

To further investigate substrate binding, we conducted 100 ns molecular dynamics (MD) simulations of the apo form and two distinct mannopentaose (M5)-bound complexes of GmMAN19-1, based on crystal structures. Simulation outcomes were evaluated using root-mean-square deviation (RMSD) and root-mean-square fluctuation (RMSF) analyses.

Throughout the simulations, the GmMAN19-1 backbone remained stable, maintaining RMSD values below 0.2 Å (Figure S9A). Both binding configurations, referred to as mode 1 (hydrolytic) and mode 2 (transglycosylation), exhibited conformational stability, with M5 RMSD values remaining below 0.3 Å (Figure S9B). In both modes, key hydrogen bonds between M5 and catalytic residues were preserved. Notably, the reducing-end disaccharide of M5 displayed greater conformational flexibility in the transglycosylation mode.

RMSF analysis revealed decreased flexibility in three regions (residues 74 to 93, 261 to 290, and 350 to 360) in the M5-bound states compared with the apo form (Figure S9C,D, highlighted in orange), suggesting that substrate binding induces local rigidification. Interestingly, binding mode 2 induced slightly more local conformational change than either mode 1 or the apo state (Figure S9C,D, highlighted in yellow). These findings support the structural feasibility of multiple productive M5-binding orientations and provide a mechanistic rationale for the enzyme’s dual catalytic activity.

### Enzymatic Kinetics of GmMAN19-1

To determine appropriate conditions for steady-state kinetic assays, time-course experiments were conducted using the DNS method. The linear phase of product formation was identified under different substrate and temperature condition (Figure S10). For LBG, steady-state was reached within 10 min at 40 °C and 15 min at 30 °C using 10 μg of enzyme (Figure S10A,B). For GG, 10 μg of enzyme was sufficient at 40 °C (20 min). whereas 20 μg was required at 30 °C to achieve measurable activity within 10 min (Figure S10C,D). Based on these results, kinetic assays were performed under the following conditions: for LBG, 5 min at 40 °C and 10 min at 30 °C; for GG, 10 min at both temperatures with approriate enzyme concentrations. These settings ensured initial rate measurements within the linear phase of the reaction.

Kinetic parameters determined with LBG revealed higher catalytic efficiency at 40 °C, with a K_M_ of 4.98 ± 0.41 mg·mL^–^^1^, *V*_max_ of 70.96 ± 2.69 U·mg^–^^1^, *k*_cat_ of 41.35 ± 1.96 s^–^^1^, and *k*_cat_/K_M_ of 8.30 ± 0.39 mL·mg^–^^1^·s^–^^1^. At 30 °C, the enzyme displayed a lower K_M_ of 2.67 ± 0.25 mg·mL^–^^1^, but also reduced *V*_max_ (18.42 ± 0.62 U·mg^–^^1^) and *k*_cat_ (13.44 ± 0.46 s^–^^1^), resulting in a *k*_cat_/K_M_ of 5.03 ± 0.17 mL·mg^–^^1^·s^–^^1^ (Figure 5A,B for LBG and Table S2).

**Figure 5:**
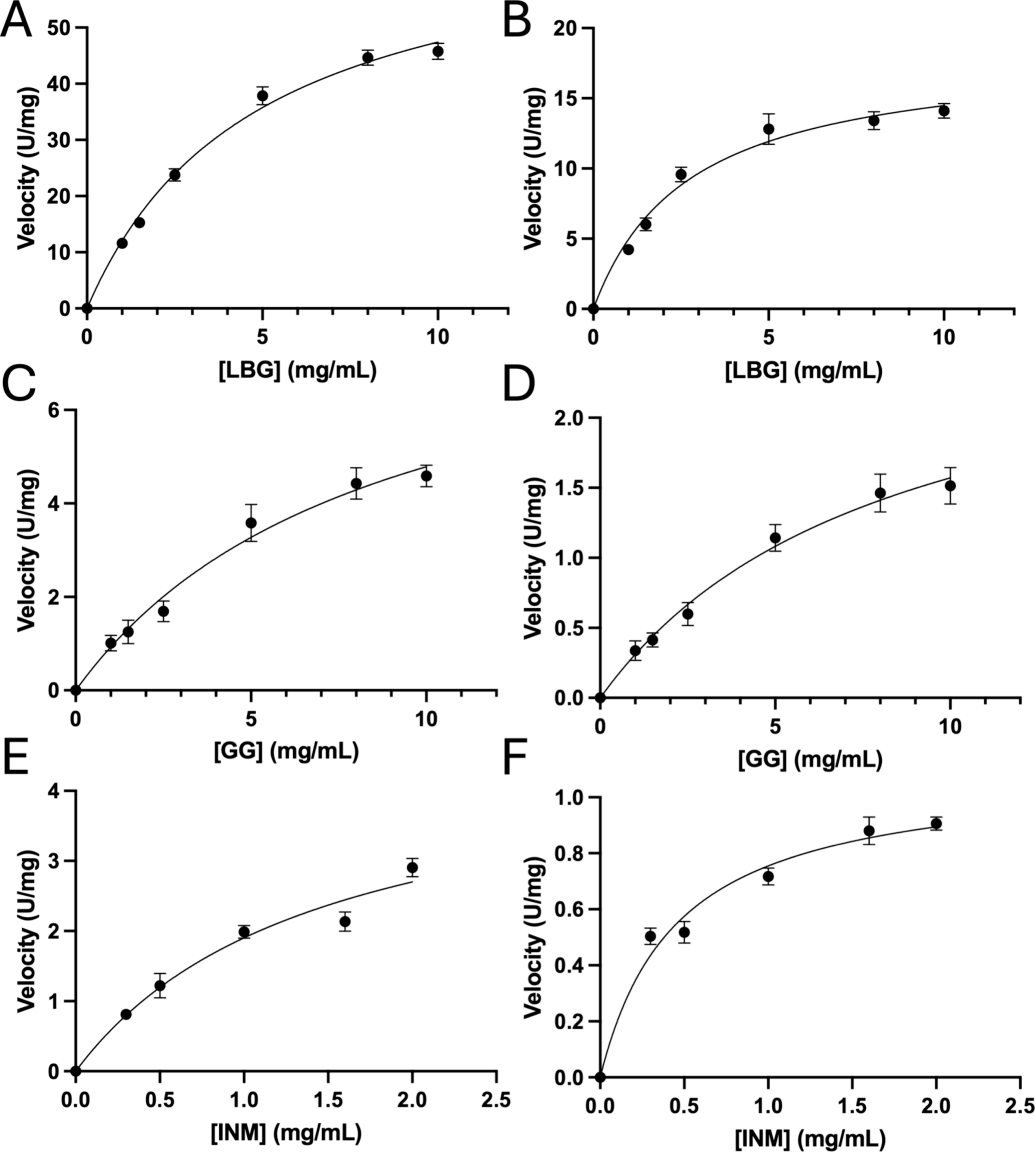
Michaelis–Menten plots of GmMAN19-1 using locust bean gum (LBG), guar gum (GG), or ivory nut mannan (INM) as the substrate. (A) Plot of GmMAN19-1 using LBG as a substrate; the measurements were performed at 40 °C for 5 min. (B) Plot of GmMAN19-1 using LBG as a substrate; the measurements were performed at 30 °C for 10 min. (C) Plot of GmMAN19-1 using GG as a substrate; the measurements were performed at 40 °C for 10 min. (D) Plot of GmMAN19-1 using GG as a substrate, the measurements were performed at 30 °C for 15 min. (E) Plot of GmMAN19-1 using INM as a substrate, the measurements were performed at 40 °C for 10 min. (F) Plot of GmMAN19-1 using GG as a substrate, the measurements were performed at 30 °C for 10 min.

When tested with the more highly branched GG substrate, GmMAN19-1 exhibited markedly lower catalytic efficiency. At 40 °C, K_M_ was 8.80 ± 1.42 mg·mL^–^^1^, *V*_max_ 9.00 ± 0.82 U·mg^–^^1^, *k*_cat_ 6.56 ± 0.60 s^–^^1^, and *k*_cat_/K_M_ 0.75 ± 0.07 mL·mg^–^^1^·s^–^^1^. At 30 °C, the corresponding values were: K_M_ 8.24 ± 1.47 mg·mL^–^^1^, *V*_max_ 2.86 ± 0.28 U·mg^–^^1^, *k*_cat_ 2.99 ± 0.21 s^–^^1^, and *k*_cat_/K_M_ 0.36 ± 0.03 mL·mg^–^^1^·s^–^^1^ (Figure 5C,D and Table S2).

We also evaluated the kinetics of GmMAN19-1 toward ivory nut mannan (INM), a linear storage mannan with minimal galactose side chains. Due to its poor solubility, INM was pretreated with 1% NaOH and sonicated to enhance solubilization. The enzyme displayed moderate activity toward INM with *V*_max_ values of 4.65 U·mg^–^^1^ and 40 °C and 1.10 U·mg^–^^1^ at 30 °C, and corresponding K_M_ values of 1.20 mg·mL^–^^1^ and 0.38 mg·mL^–^^1^, respectively (Figure S10E,F and Figure 5E,F)

Together, these data indicate that GmMAN19-1 preferentially hydrolyzes substrates with lower degrees of galactose side-chain substitution, such as LBG and INM, over more extensively branched substrates like GG. This substrate preference aligns with its proposed physiological role in the degradation of storage polysaccharides during seed gemination.

### Site-Directed Mutagenesis Validates Catalytic and Substrate-Binding Residues

To further verify the catalytic residues and investigate the structural basis of substrate recognition, we performed site-directed mutagenesis of E186 (putative acid/base) and Q267, a substrate-binding residue at the +1 subsite exhibiting sequence variability among GH5 β-mannanases (Figure S11A). Structural alignment revealed that Q267 resides in a flexible loop adjacent to the +1 subsite, potentially impacting steric accommodation of branched substrates (Figure S11B). To ensure that the mutations did not disrupt protein folding, we analyzed all variants by circular dichroism spectroscopy. The CD spectra of E186A and Q267W were nearly identical to wild-type GmMAN19-1 at both pH 4.5 and 7.5, confirming proper structural integrity (Figure S12). Q267 was substituted with tryptophan to examine the effects of enhanced hydrophobic packing and potential π–π stacking with the sugar ring. This substitution was designed to test whether the bulky aromatic side chain of Trp could enhance substrate interaction.

Further analysis of the Michaelis–Menten plots (Figure S13) revealed condition-dependent changes in the kinetic profile of Q267W. At 40 °C, Q267W exhibited an elevated Vmax toward both LBG (70.74 U·mg^–^^1^) and GG (13.64 U·mg^–^^1^) compared to wild-type. However, this was accompanied by increased K_M_ values, especially notable for GG (12.68 mg·mL^–^^1^ vs 4.55 mg·mL^–^^1^), suggesting reduced substrate affinity despite enhanced catalytic rate. At 30 °C, the effect persisted with slightly lower Vmax and even higher K_M_, particularly for GG (14.24 mg·mL^–^^1^). These results indicate that Q267 modulates substrate accommodation and catalytic turnover, especially in the context of sterically hindered substrates like GG (Figure S11B and Table S2).

Furthermore, the Q267W mutant displayed an altered transglycosylation profile, generating shorter-chain oligosaccharides more prominently than the wild-type enzyme (Figure S3). In contrast, the E186A mutant showed no detectable hydrolytic or transglycosylation activity under any tested conditions, confirming its essential catalytic role. The mechanistic divergence between hydrolysis and transglycosylation arises from the identity of the nucleophile that attacks the glycosyl-enzyme intermediate. In hydrolysis, the nucleophile is a water molecule, whereas in transglycosylation, it is typically the hydroxyl group of an acceptor sugar. Although both reactions follow a conserved retaining double-displacement mechanism, the ability of the active site to accommodate either water or a sugar acceptor plays a critical role in determining the reaction outcome. In this context, Q267W likely shifts the balance toward transglycosylation by enhancing the binding of acceptor sugars at the +1 and +2 subsites. These results validate the dual functions of Q267 in substrate positioning and product specificity, and highlight the essential role of E186 in acid/base catalysis.

### Comparison of β-Mannanase Activity with Plant and Fungal Homologues

The enzymatic properties of GmMAN19-1 were compared with those of β-mannanases derived from other plant and fungal sources (Table S3). GmMAN19-1 displayed optimal reaction conditions (pH 4.6 and 40 °C) that were largely consistent with those reported for other plant β-mannanases, suggesting conservation of catalytic traits among plant homologues. In terms of specific activity, GmMAN19-1 exhibited relatively strong performance, positioning it among the more active enzymes in the plant group. By contrast, β-mannanases from fungal sources demonstrated markedly different biochemical characteristics. These fungal enzymes typically display lower optimal pH values and higher temperature optima, often exceeding 50 °C, and exhibit substantially higher specific activities.^32,33^ Such distinctions point toward intrinsic structural differences that may underlie enhanced thermal stability and catalytic efficiency in fungal β-mannanases (Table S3). These findings underscore the influence of evolutionary origin on β-mannanase function and highlight the potential of structural studies to uncover molecular features that account for the differences in stability, substrate preference, and catalytic performance across diverse taxa.

## Discussion

### Functional Role of GmMAN19-1 in Soybean

GmMAN19-1 is predominantly expressed in cotyledon tissues beginning at 7 days after germination, whereas GmMAN11 expression peaks during the first day. This temporal divergence suggests that GmMAN11 functions in the early phases of germination, while GmMAN19-1 operates later during radicle elongation and seedling establishment. Given that cotyledons are a principal reservoir of storage polysaccharides in leguminous plants, GmMAN19-1 likely contributes to the mobilization of these carbohydrates to support postgerminative growth. By hydrolyzing mannan-based polysaccharides into oligosaccharides or disaccharides, the enzyme helps sustain energy supply during periods of active development. In addition, GmMAN19-1 may be involved in polysaccharide recycling or repolymerization under conditions of reduced energy demand, contributing to carbohydrate homeostasis. The distinct expression profiles and catalytic properties of GmMAN11 and GmMAN19-1 thus reflect a functional differentiation that enables the soybean to adaptively regulate carbohydrate metabolism throughout germination and early development.

### Substrate Preference and Kinetic Behavior

Kinetic analysis revealed that GmMAN19-1 exhibits higher catalytic efficiency toward linear mannans such as LBG compared to highly branched substrates like GG. This preference aligns with its physiological role in degrading storage polysaccharides in cotyledons. The Q267W mutant showed enhanced turnover (*V*_max)_ toward GG, suggesting a broader or more flexible substrate-binding profile. This functional enhancement is consistent with structural comparisons showing that Q267 lies in a variable loop region across GH5 β-mannanases (Figure S11B), where side chain orientation may influence steric compatibility with branched substrates such as GG.

### Structural Features and Substrate Recognition in GmMAN19-1

Distortion of the sugar ring at the −1 subsite is a recurring feature in glycoside hydrolases and is crucial for catalysis.^34^ This conformational change bring the glycosidic bond into proximity with catalytic residues and reorients hydroxyl groups to reduce steric hindrance.^35^ In β-mannanases, mannose at the −1 position typically undergoes a conformational itinerary from ^1^S_5_ to B_2,5_ and ultimately returns to O_5_^2^. In the GmMAN19-1/M5 substrate complex, the mannose at the −1 subsite adopts a B_2,5_ skew-boat conformation. Structural analysis shows that this distortion aligns well with the orientation of the aromatic residues Y264, W344, and Y358, which form a hydrophobic platform stabilizing the distorted sugar. The aromatic residue Y264, highly conserved across species (Figure S14A), is positioned within the hydrophobic core near the glycosidic bond, suggesting a key role in stabilizing sugar distortion (Figure S14B,C). Together with N185 at the −1 site and W118, Q267, and E223 at the +1 site, these residues orchestrate hydrogen bonding and stacking interactions across subsites −5 to +2, ensuring proper substrate preorganization and catalysis.

Hydrogen bonding and stacking interactions extend across the binding cleft from subsites −5 to +2. At subsite −1, N185 stabilizes the C2-OH group, while W118, Q267, and E223 contribute to recognition at the +1 site. The cumulative interactions from aromatic and polar residues ensure precise orientation of mannooligosaccharides for catalysis.

### Accommodation of the Branching Chains of Galactomannan

Galactomannans possess galactose side chains linked via α-1,6 glycosidic bonds to the C6 position of mannose units. In the GmMAN19-1/M5 complex, C6-OH groups at subsites +2, −1, and −5 project outward from the binding groove, offering spatial flexibility for accommodating α-1,6-linked galactose moieties (Figure S15). This structural arrangement suggests that GmMAN19-1 can effectively process branched galactomannans, particularly where steric hindrance is minimal, such as at subsites +2, −3, and −5. This tolerance toward branching may enhance the enzyme’s adaptability toward natural polysaccharide substrates in seeds.

### Catalytic Mechanism and Validation by Mutagenesis

Site-directed mutagenesis validated the catalytic roles of key residues. E186A abolished activity, confirming its function as the general acid/base. The Q267W mutation altered substrate recognition at the +1 site, increasing Vmax for GG while slightly reducing substrate affinity. These findings support the role of Q267 in modulating substrate accommodation and product distribution, particularly under transglycosylation-favorable conditions.

### Structural Relatives and Evolutionary Divergence

Structural comparison using the DALI server^36^ revealed that GmMAN19-1 shares significant similarity with several β-mannanases from both plant and fungal sources (Table S4), including LeMAN4 from *Lycopersicon esculentum*,^24^ RmMAN5A from *Rhizomucor miehei*,^37^ CsMAN5A from *Chrysonilia sitophila*,^38^ PaMAN5A from *Podospora* anserina,^39^ TrMAN from *Trichoderma reesei*,^40^ and TpMAN from *Thermotoga petrophila*.^41^ While all these enzymes belong to GH5 and share the canonical (α/β)_8_ TIM barrel fold, notable divergence exists at the negative subsites of their active sites. For example, TpMAN features a long and narrow groove, favoring linear oligosaccharides, whereas CsMAN5A exhibits loop insertions that block access beyond the −2 subsite, restricting its function to exo-β-mannanase activity. In contrast, plant-derived enzymes such as GmMAN19-1 and LeMAN4 possess shallow, narrower grooves at the negative subsites, which likely modulate their substrate specificity and endoactivity profiles (Figure S16).

While GH5 mannanases such as GmMAN19-1 adopt a (β/α)_8_-barrel fold, GH11 xylanases utilize a β-jelly roll architecture. Despite these structural differences, both enzyme classes share a common double-displacement mechanism involving a glycosyl-enzyme intermediate, with conserved general acid/base and nucleophilic residues. This illustrates how similar catalytic outcomes can arise through distinct structure solutions, understanding the functional convergence of glycoside hydrolases under evolutionary pressure. Such mechanistic parallels provide a framework for engineering substrate specificity or stability in diverse GH enzymes.^42,43^

GmMAN19-1 exhibits a developmentally regulated expression pattern and displays acid-stable, moderately thermosensitive enzymatic properties consistent with its role in postgermination energy mobilization. Structural analysis reveals a canonical GH5 fold with specific adaptations at the active site, including stabilization of a distorted −1 subsite and tolerance for branched galactomannans. These features highlight GmMAN19-1 as a promising candidate for applications in food processing, biomass conversion, and industrial biotechnology, particularly under acidic conditions and with sterically hindered substrates.

## Supplementary Material



## Notes

Table S1: soybean β-mannanase sequences identified through genome mining; Table S2: kinetic parameters of GmMAN19-1 and its mutants toward LBG and GG; Table S3: comparison of GmMAN19-1 with the activity of other β-mannanases; Table S4: structural comparison of GmMAN19-1 with structural relatives; Figure S1: molecular characterization of GmMAN19-1; Figure S2: SDS-PAGE analysis of GmMAN19-1; Figure S3: transglycosylation activities of GmMAN19-1 and GmMAN19-1-Q267W; Figure S4: manno-oligosaccharide product analysis by HPAEC-PAD; Figure S5: mass spectrometric analysis of mannooligosaccharides; Figure S6: X-ray diffraction pattern of GmMAN19-1 crystal; Figure S7: Fo–Fc omit maps of mannopentaose bound to GmMAN19-1; Figure S8: protein–ligand interaction LigPlot of GmMAN19-1 and mannopetaose; Figure S9: molecular simulation and ligand-induced conformational effects of GmMAN19-1; Figure S10: presteady-state kinetic analysis of GmMAN19-1; Figure S11: sequence conservation and structural variation at residue Q267 across GH5 β-mannanases; Figure S12: CD spectra of GmMAN19-1 and its mutants at different pH conditions; Figure S13: Michaelis–Menten plots of Q267W enzyme; Figure S14: Y264 is highly conserved and aids sugar torsion at the −1 subsite; Figure S15: accommodation of galactose side chain at surface binding site; Figure S16: substrate binding cleft differences between GmMAN19-1 and six related β-mannanases (PDF)

## Notes

Final coordinates and structure factors for GmMAN19-1 in apo form and in complex with mannopentaose have been deposited in the protein data bank (PDB) with the accession code 9M20 and 9M21, respectively.
