# Supplementary material for: Unveiling the Structural and Biochemical Characteristics of an Acidophilic β‑Mannanase from Soybean (Glycine max)
Source: J Agric Food Chem. 2025 Sep 25;73(40):25479–90. doi: 10.1021/acs.jafc.5c03141 (PMC12512182; doi:10.1021/acs.jafc.5c03141)
Supplement: Supplementary file 1 [file jf5c03141_si_001.pdf]

Supporting Information

Unveiling the Structural and Biochemical Characteristics of an Acidophilic β-Mannanase from Soybean (Glycine max)

Chun-Jung Lin[a], Chao-Cheng Cho[a], Sheng-Chia Chen[a], Gloria Meng-Hsuan Lin[a], Cheng-Yang Huang[a], and Chun-Hua Hsu[a,b,c,d*]

[a]Department of Agricultural Chemistry, National Taiwan University, Taipei 10617, Taiwan

[b]Institute of Biochemical Sciences, National Taiwan University, Taipei 10617, Taiwan

[c]Genome and Systems Biology Degree Program, National Taiwan University and Academia Sinica, Taipei 10617, Taiwan

[d]Center for Computational and Systems Biology, National Taiwan University, Taipei 10617, Taiwan

*Corresponding authors

Chun-Hsu Hsu

Email: andyhsu@ntu.edu.tw; Tel: +886-2-33664468

Table S1. A complete list of the 21 soybean *β*-mannanase sequences identified through genome mining.

Abbreviation	Phyozome v12.0 code	UniProtKB
GmMAN1	Glyma01g171900	I1J8S1
GmMAN3-1	Glyma03g002100	I1JK01
GmMAN3-2	Glyma03g226000	Q0PKY2
GmMAN3-3	Glyma03g229100	I1JK28
GmMAN4	Glyma04g030400	I1JT90
GmMAN6-1	Glyma06g030500	I1K7Q5
GmMAN6-2	Glyma06g292400	I1KF18
GmMAN9-1	Glyma09g224500	I1L5I1
GmMAN9-2	Glyma09g273500	I1L6Z0
GmMAN10	Glyma10G122000	K7LIY0
GmMAN11	Glyma11g071300	I1LHV7
GmMAN12-1	Glyma12g012500	I1LP13
GmMAN12-2	Glyma12g113400	I1LS27
GmMAN12-3	Glyma12g204700	I1LUH0
GmMAN13	Glyma13g296600	I1M3S5
GmMAN14	Glyma14g072600	I1M8A4
GmMAN16	Glyma16g220300	K7MJ31
GmMAN17	Glyma17g252400	I1MXX7
GmMAN18	Glyma18g215600	I1N3A5
GmMAN19-1	Glyma19g41090 (Glyma19G22300)	I1NBK5
GmMAN19-2	Glyma19g41410 (Glyma19g226300)	K7MZS1

Table S2. Kinetic parameters of GmMAN19-1 and its mutants toward LBG and GG.

Enzyme		Temp.	K_M ($mg\cdot mL^{-1}$)	V_{max} (U/mg)	k_{cat} (s^{-1})	k_{cat}/K_M ($mL\cdot mg^{-1}\cdot s^{-1}$)
WT	LBG	40℃	4.98±0.41	70.96±2.69	41.35±1.96	8.30±0.39
Q267W	LBG	40℃	6.07±0.76	70.74±4.35	41.23±3.18	6.79±0.52
E186A	LBG	40℃	ND	ND	ND	ND
WT	LBG	30℃	2.67±0.25	18.42±0.62	13.44±0.46	5.03±0.17
Q267W	LBG	30℃	3.80±0.61	24.91±1.65	18.18±1.21	4.78±0.32
E186A	LBG	30℃	ND	ND	ND	ND
WT	GG	40℃	8.80±1.42	9.00±0.82	6.56±0.60	0.75±0.07
Q267W	GG	40℃	12.68±1.60	13.64±1.10	9.94±0.80	0.78±0.06
E186A	GG	40℃	ND	ND	ND	ND
WT	GG	30℃	8.24±1.47	2.86±0.28	2.99±0.21	0.36±0.03
Q267W	GG	30℃	14.24±2.28	5.99±0.64	4.37±0.46	0.30±0.03
E186A	GG	30℃	ND	ND	ND	ND

ND: Not detected.

Table S3. Comparison of GmMAN19-1 with the activity of other β-mannanases.

Protein	Optimal		LBG				Ref.
	pH	T_{opt} (℃)	K_M (mg·mL^{-1})	V_{max} (U/mg)	k_{cat} (s^{-1})	k_{cat}/K_M (mL·mg^{-1}·s^{-1})	
Source from Plant							
GmMAN19-1	4.6	40	4.98	70.96	41.35	8.30	This study
GmMAN19-1	4.6	30	2.67	18.42	13.44	5.03	This study
AtMAN5-1e (*Arabidopsis thaliana)*	5.0	35	7.4	65.4	52.69	7.12	(Wang et al., 2014b)
AtMAN5-2 (*Arabidopsis thaliana)*	5.0	25	12.8	2.5	2.1	0.20	(Wang et al., 2015)
Source from Fungi							
AnMan5A (*Aspergillus niger*)	3.5	70	1.09	221.8	231.1	210.1	(Li et al., 2012)
ThMan5A (*Trichodermaharzianum*)	5.5	70	3.99	1544	3083	771	(Wang et al., 2014a)
AsMan5A *(Aspergillus sulphureus)*	2.4	50	0.92	366	276	297	(Chen et al., 2007)
AfMAN1 *(Aspergillus fumigatus)*	4.5	60	3.07	562	1935	630	(Puchart et al., 2004)
ManBK *(Aspergillus niger)*	5.3	50	0.79	643	180	2279	(Huang et al., 2014)

Table S4. Structural comparison of GmMAN19-1 with its six closest protein structure identified using the DALI server.

No	Structural comparison	DALI Z-score	RMSD	%id	Species source
A	*Glycine max* MAN19-1	—	—	—	Plant
B	*Lycopersiconesculentum* β-mannanase 4a (LeMAN) (PDB code : 1RH9)	59.7	1.0	60	Plant
C	*Rhizomucormiehei* β-mannanase (RmMAN5A) (PDB code : 4QP0)	46.8	1.8	32	Fungi
D	*ThermotogapetrophilaRKU-1* βmannanase (TpMAN) (PDB code : 6TNT)	45.1	1.6	34	Bacteria
E	*Podosporaanserina* mannanases (PaMAN5A) (PDB code : 3ZIZ)	44.0	2.0	31	Fungi
F	*Trichodermareesei* β-mannanase (TrMAN) (PDB code : 1QNO)	43.1	1.9	32	Fungi
G	*Chrysonilia sitophila* β-mannanase (CsMAN5A) (PDB code : 4AWE)	44.6	2.1	32	Fungi

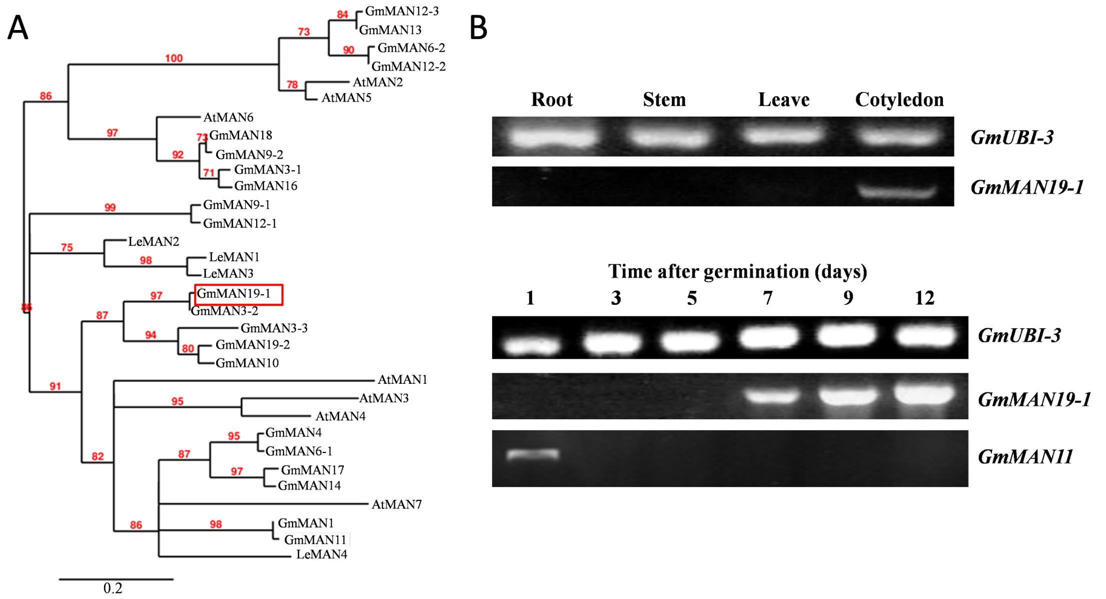

Figure S1. Molecular characterization of GmMAN19-1.

(A) Phylogenetic tree of 21 soybean β-mannanases and β-mannanases from other plants. All sequences beginning with "Gm" represent soybean (Glycine max) β-mannanases. Other known plant β-mannanases include LeMAN1 (AAB87859), LeMAN2 (AAG00315.2), LeMAN3 (AAG14352), and LeMAN4a (AAK97760) from tomato (Lycopersicon esculentum), as well as AtMAN1 (NP_171733.2), AtMAN2 (NP_179660.2), AtMAN3 (NP_187700.1), AtMAN4 (NP_187701.1), AtMAN5 (NP_194561.1), AtMAN6 (NP_195813.1), and AtMAN7 (NP_201447) from Arabidopsis thaliana. (B) Expression of GmMAN19-1 and GmMAN11 in various soybean tissues and during germination. Primers for soybean ubiquitin 3 (GmUBI-3) were used as an internal control to ensure equal cDNA template concentrations across samples. Upper panel: GmMAN19-1 expression in various tissues of 12-day-old soybean plants. GmMAN19-1 is exclusively expressed in cotyledons, with no detectable expression in other tissues. Lower panel: Expression of GmMAN19-1 and GmMAN11 in cotyledons at different germination time points. GmMAN19-1 expression is first detected on day 7 after germination and gradually increases over time, whereas GmMAN11 is weakly expressed at an earlier stage (day 1), suggesting distinct roles in germination and seedling development.

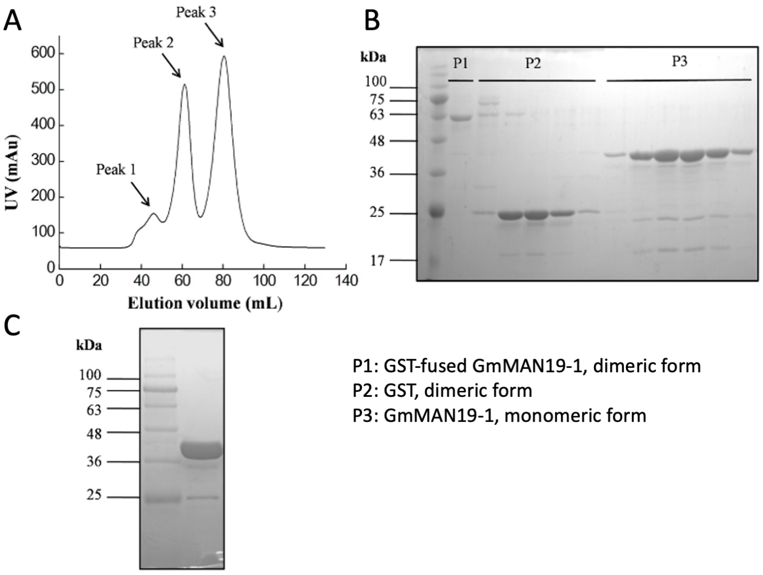

Figure S2. Separation of GmMAN19-1 and GST-tag by gel filtration chromatography.

(A) Elution profile showing separation of GmMAN19-1 and GST-tag using gel filtration chromatography. (B) SDS-PAGE analysis of fractions corresponding to Peak 1 (P1), Peak 2 (P2), and Peak 3 (P3) as indicated in panel A. (C) SDS-PAGE analysis of the final concentrated protein sample, containing approximately 10 mg/mL of purified GmMAN19-1.

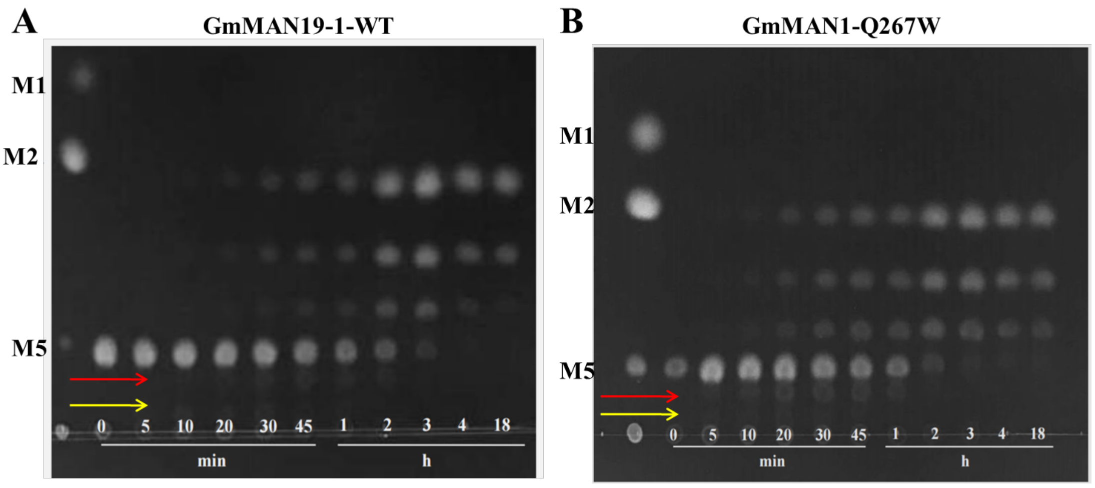

Figure S3. Transglycosylation activities of GmMAN19-1 and GmMAN19-1-Q267W.

(A) The transglycosylation activity of GmMAN19-1 using M5 as both donor and acceptor. GmMAN19-1 (1.64 µM) was incubated with 25 mM M5 in McIlvaine buffer (pH 4.6) at 40 °C for various times. (B) The transglycosylation activity of GmMAN19-1-Q267W using M5 as both donor and acceptor. GmMAN19-1-Q267W (1.64 µM) was incubated with M5 in 50 mM McIlvaine buffer (pH 4.6) at 40 °C for various times. Red arrows indicate oligosaccharide products with smaller molecular weights; yellow arrows indicate newly generated transglycosylation products.

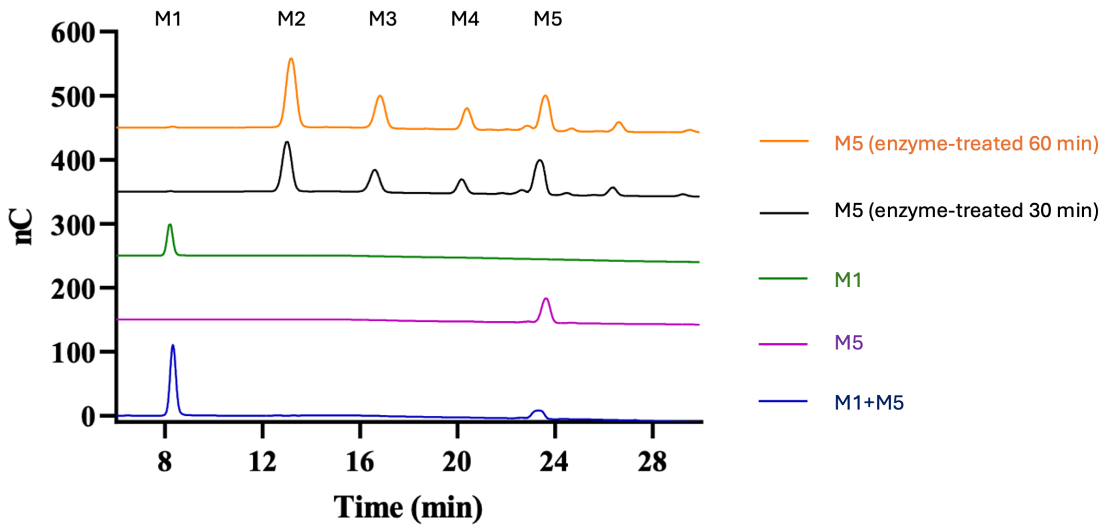

Figure S4. Manno-oligosaccharide product analysis by HPAEC-PAD after enzymatic hydrolysis of mannopentaose (M5) by GmMAN19-1.

Chromatograms show the hydrolysis products analyzed using high-performance anion-exchange chromatography with pulsed amperometric detection (HPAEC-PAD). Identified peaks correspond to mannose (M1), mannobiose (M2), mannotriose (M3), mannotetraose (M4), and the remaining mannopentaose (M5), indicating the cleavage pattern of GmMAN19-1 toward linear manno-oligosaccharides.

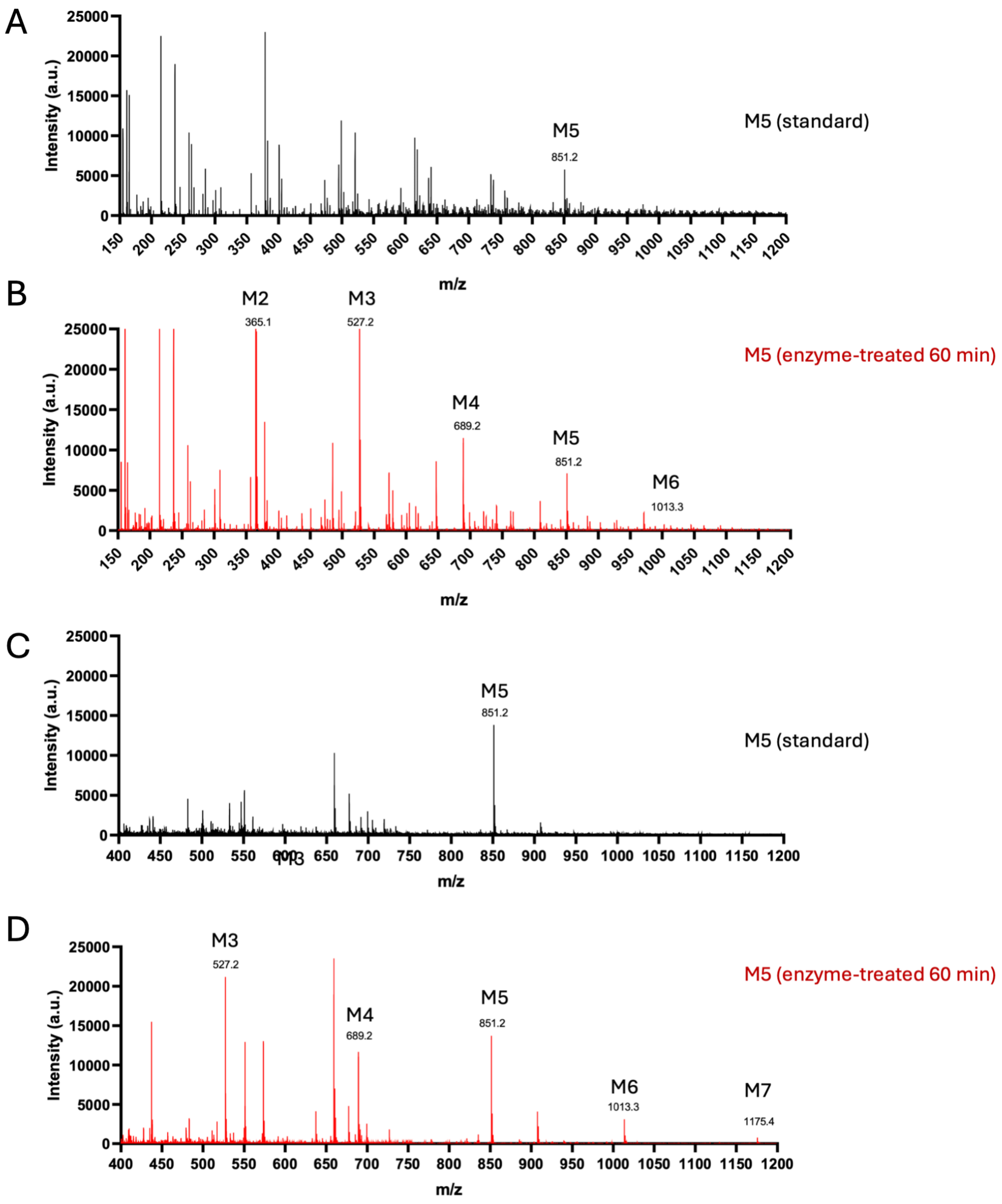

Figure S5. Mass spectrometric analysis of mannooligosaccharides.

(A) ESI–TOF mass spectrum of the mannopentaose (M5) standard. (B) ESI–TOF mass spectrum of the M5 reaction mixture with GmMAN19-1, showing ions corresponding to potential hydrolysis and/or transglycosylation products. (C) MALDI–TOF mass spectrum of the M5 standard. (D) MALDI–TOF mass spectrum of the enzymatic reaction mixture, highlighting detected product ions. Mannooligosaccharides are labeled as follows: mannobiose (M2), mannotriose (M3), mannotetraose (M4), mannopentaose (M5), mannohexaose (M6), and mannoheptaose (M7). Unless otherwise indicated, peaks are assigned as $[M + Na]^+$.

x-axis: mass-to-charge ratio (m/z); *y*-axis: relative intensity (a.u.).

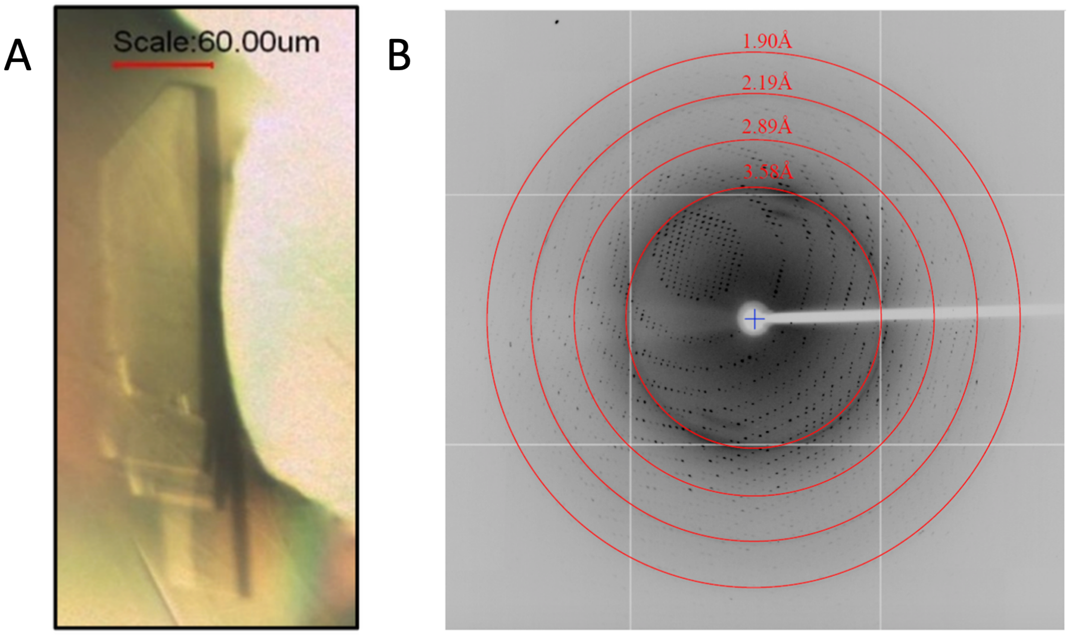

Figure S6. Protein crystal and the X-ray diffraction pattern of GmMAN19-1 crystal.

(A) Crystals of GmMAN19-1 were grown in refined condition of Crystal Screen 1-10 (0.1 M ammonium acetate, 0.1 M sodium citrate trihydrate pH4.6, 30% PEG 4000) with 0.1 M maltose and 0.1 M TECP additives. (B) Diffraction pattern was collected from GmMAN19-1 crystal on the BL13B1 beamline at NSRRC. Resolution arcs are indicated in Å (red circles).

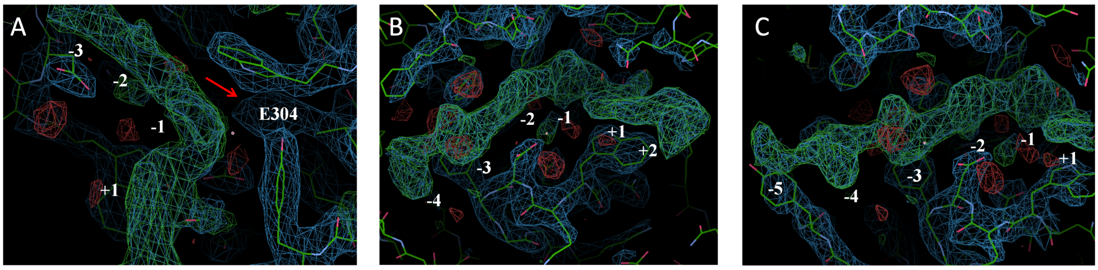

Figure S7. Real-space averaged Fo–Fc omit maps (green mesh) of mannopentaose bound to the active site residues of GmMAN19-1.

The 2Fo–Fc electron density map is shown at the 1σ level (blue), while the Fo–Fc electron density is contoured at the 3σ level, with positive density in green and negative density in red. Carbon, oxygen, and nitrogen atoms are represented as lines and are colored green, red, and blue, respectively. (A) The electron density map of the nucleophilic catalytic residue E304 shows no direct connection to mannopentaose. (B) and (C) The mannopentaose-binding region in the GmMAN19-1/M5 complex spans subsites -5 to +2.

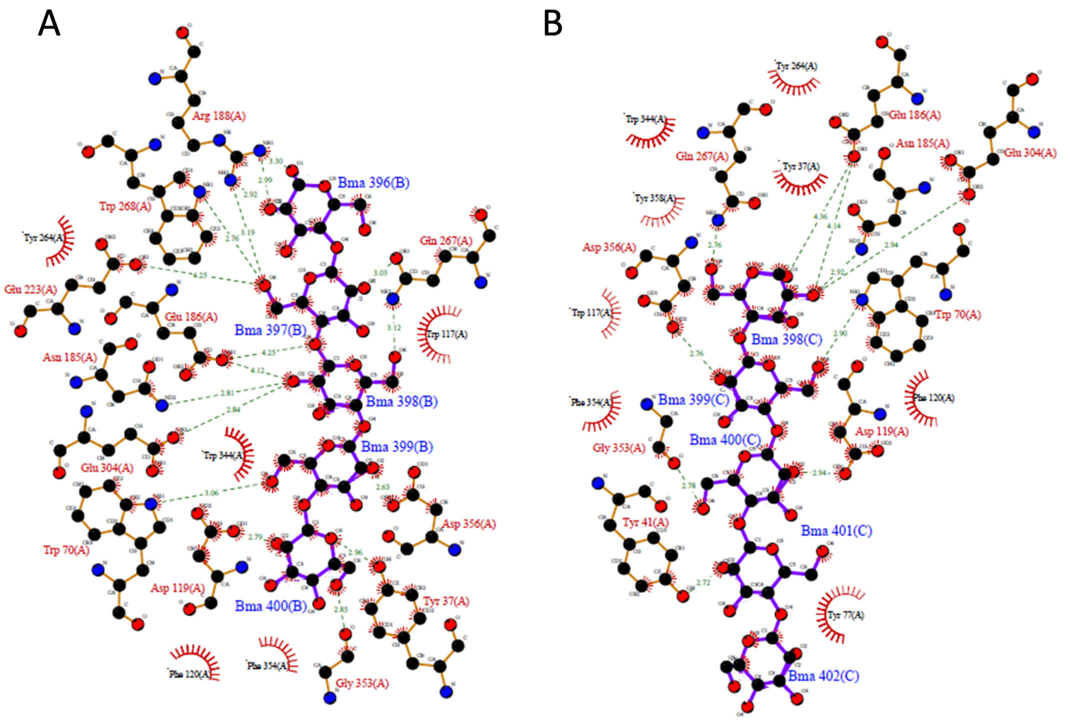

Figure S8. Protein–ligand interaction 2D map of GmMAN19-1 and mannopetaose using LIGPLOT program.

Covalent bonds of mannopentaose are in purple, covalent bonds of amino acids in light brown, hydrogen bonds are in green dashed lines. Protein residues in hydrophobic contacts with mannopentaose are represented by red semi-circles with radiating spokes. The complex structure existed two distinct binding modes of mannopentaose, one is glycohydrolase binding mode (A) and the other is transglycosylase binding mode (B).

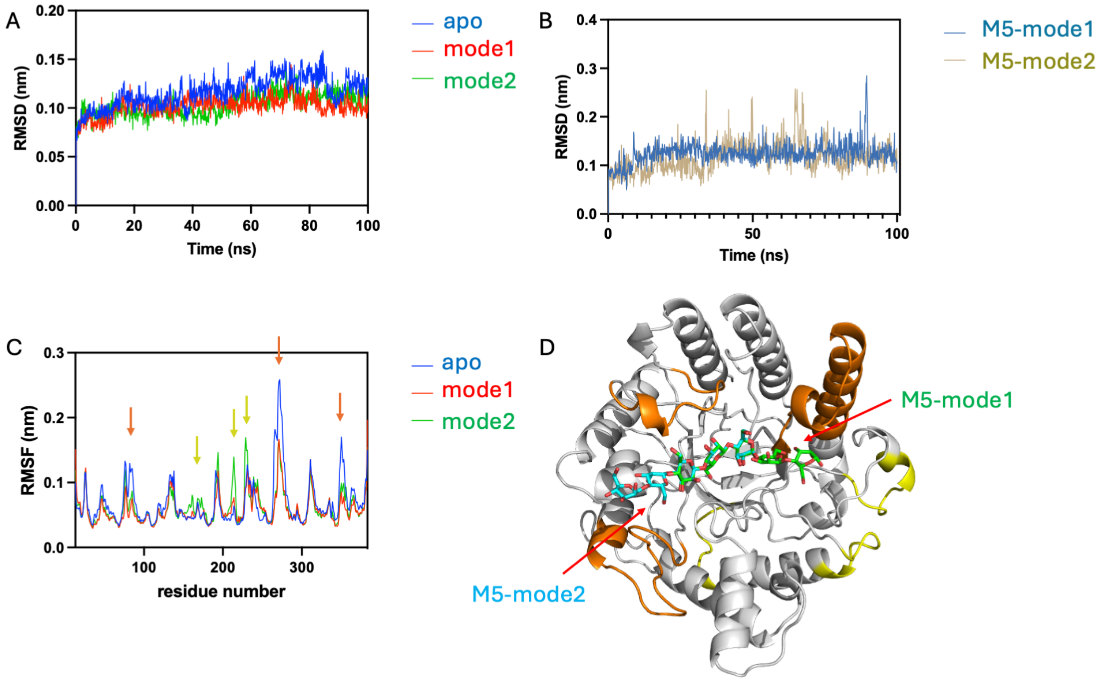

Figure S9. Molecular simulation and ligand-induced conformational effects of GmMAN19-1.

(A) Root-mean-square deviation (RMSD) of backbone atoms during 100 ns molecular dynamics (MD) simulations for the apo form and mannopentaose (M5)-bound complexes (mode 1 and mode 2). (B) RMSD of mannopentaose (M5) during simulations for mode 1 and mode 2 binding poses. (C) Root-mean-square fluctuation (RMSF) of Cα atoms in GmMAN19-1 for the apo and M5-bound states, highlighting residue-level flexibility. Regions with increased fluctuation in the apo form are indicated by an orange arrow, while regions with enhanced flexibility in the mode 2 complex are marked by a yellow arrow. (D) Ribbon representation of GmMAN19-1. M5 binding poses for mode 1 (green) and mode 2 (cyan) are shown as stick models. Flexible regions in the apo form are highlighted in orange, and a conformationally responsive region observed in the mode 1 complex is colored yellow.

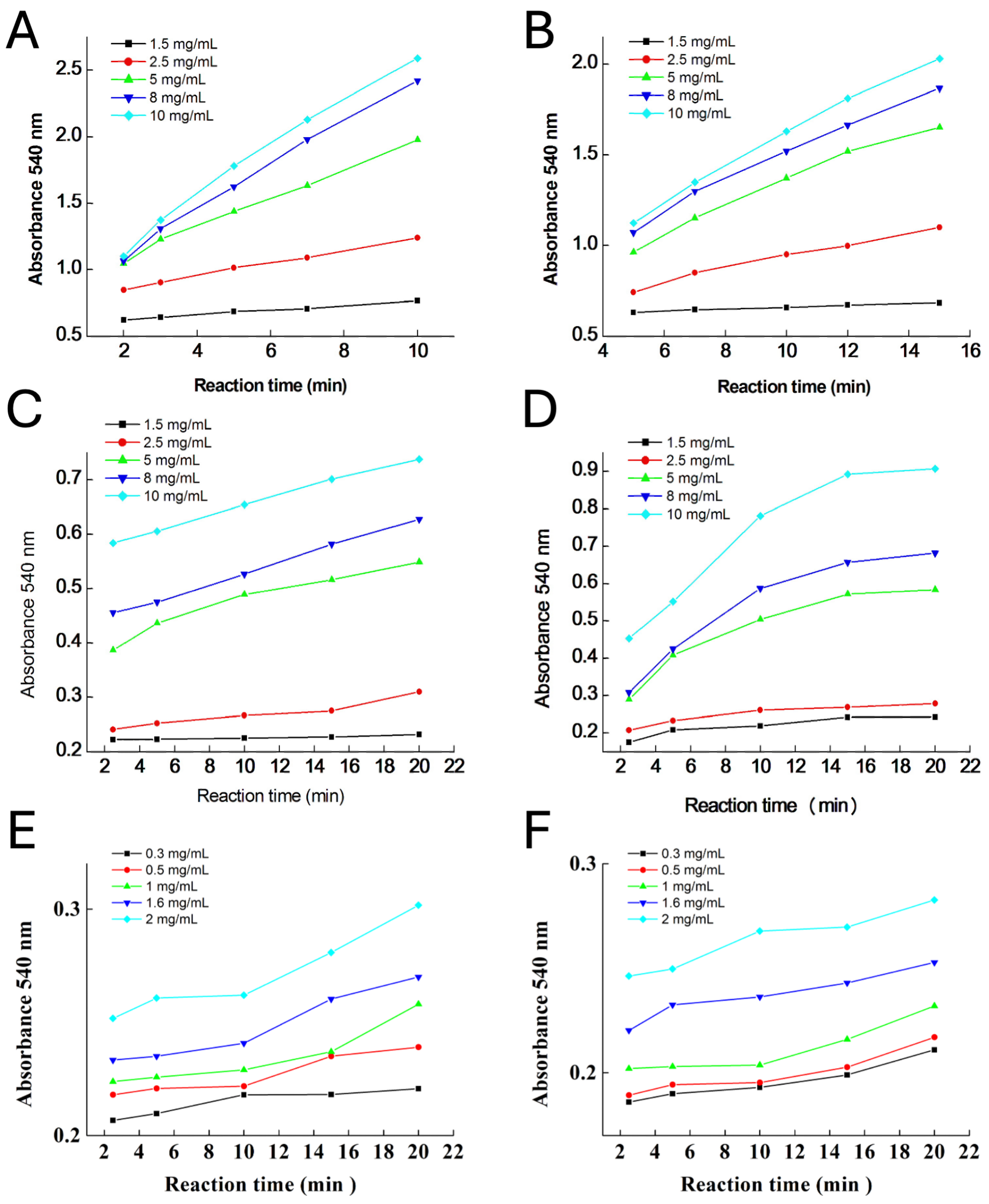

Figure S10. Pre-steady-state kinetic analysis of GmMAN19-1.

(A) Hydrolysis of LBG at varying substrate concentrations in McIlvaine buffer (pH 4.6) at 40°C. (B) Hydrolysis of LBG at varying substrate concentrations in McIlvaine buffer (pH 4.6) at 30°C. (C) Hydrolysis of GG at varying substrate concentrations in McIlvaine buffer (pH 4.6) at 40°C. (D) Hydrolysis of GG at varying substrate concentrations in McIlvaine buffer (pH 4.6) at 30°C. (E) and (F)INM

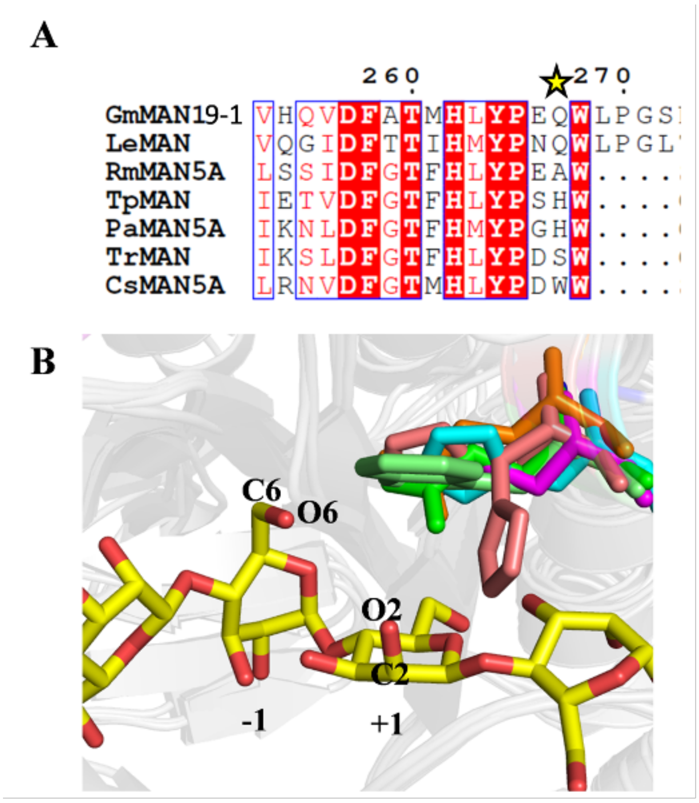

Figure S11. Sequence conservation and structural variation at residue Q267 across GH5 β-mannanases.

(A) Structure-based multiple sequence alignment of GmMAN19-1 with selected GH5 β-mannanases. The position of Q267 is marked by a star, showing moderate conservation and variability across species. (B) Structural superposition of GmMAN19-1 and six homologous β-mannanase structures at the Q267-equivalent position. Amino acid residues at this site are shown in stick representation, highlighting variations in side-chain composition and orientation. The overlaid structures include GmMAN19-1 (green), LeMAN (orange), RmMAN (blue), TpMAN (deep salmon), PaMAN (cyan), TrMAN (magenta), and CsMAN5A (lime green). Mannopentaose is shown as a yellow stick model to illustrate the spatial relationship between Q267 and the +1 subsite sugar.

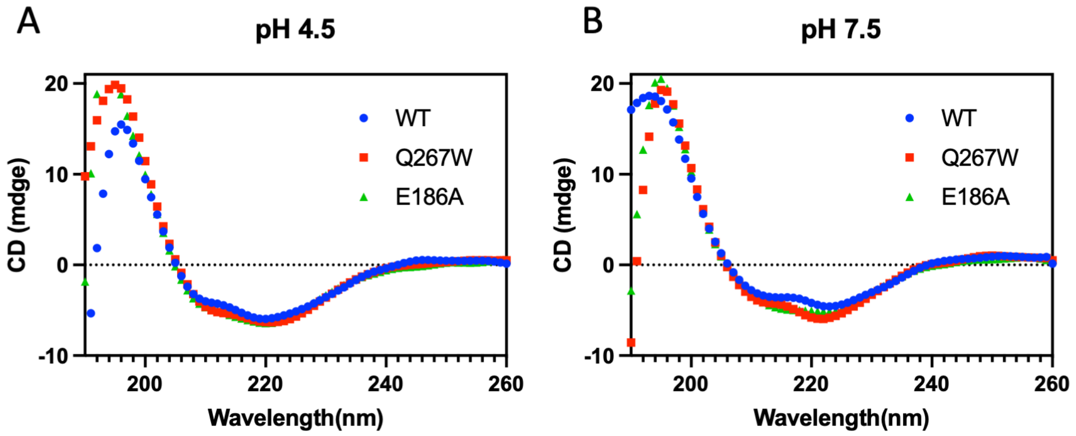

Figure S12. CD spectra of GmMAN19-1 and its mutants at different pH conditions. (A) CD spectra of GmMAN19-1 and its mutants (Q267W, E186A) at pH 4.5. (B) CD spectra of GmMAN19-1 and its mutants (Q267W, E186A) at pH 7.5. The CD profiles coincide under both conditions, suggesting that the mutations do not alter the overall secondary structure.

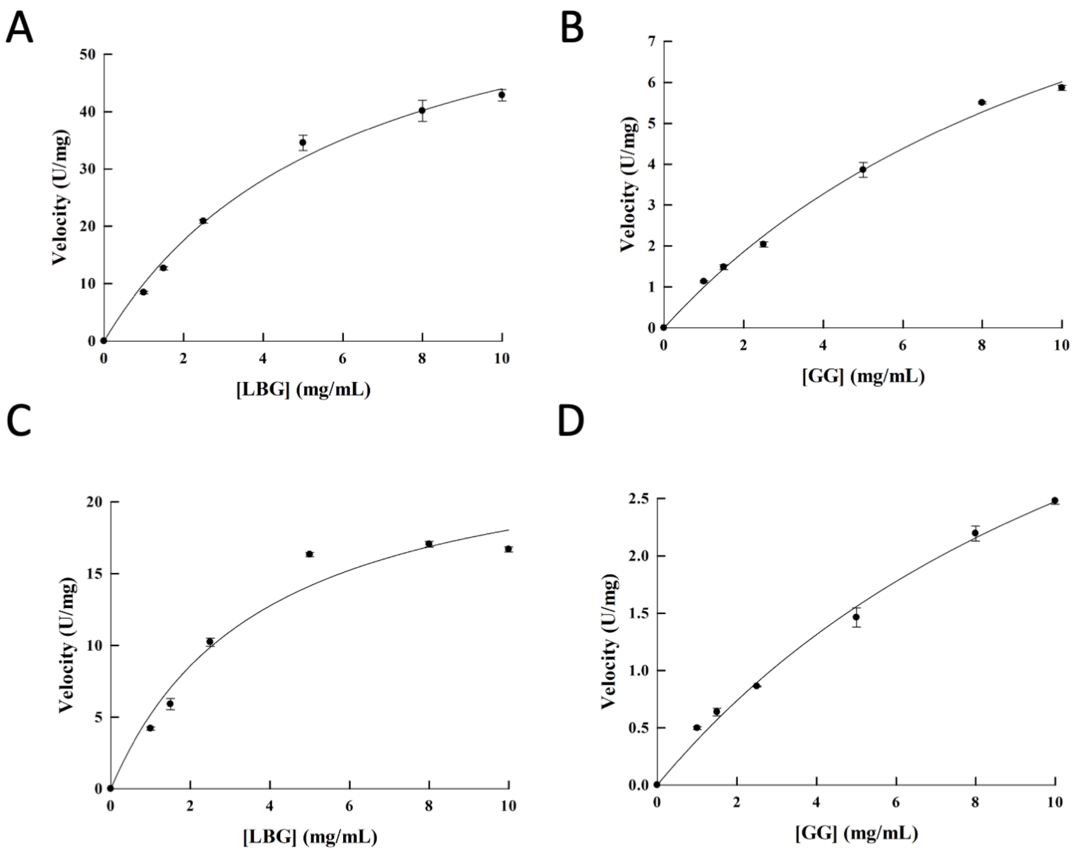

Figure S13. Michaelis-Menten plots of Q267W using locust bean gum (LBG) or guar gum (GG) as substrate.

(A) The plot of Q267W using LBG as substrate, measured at 40 °C for 5 min. (B) The plot of Q267W using GG as substrate, measured at 40 °C for 10 min. (C) The plot of Q267W using LBG as substrate, measured at 30 °C for 10 min. (D) The plot of Q267W using GG as substrate, measured at 30 °C for 15 min.

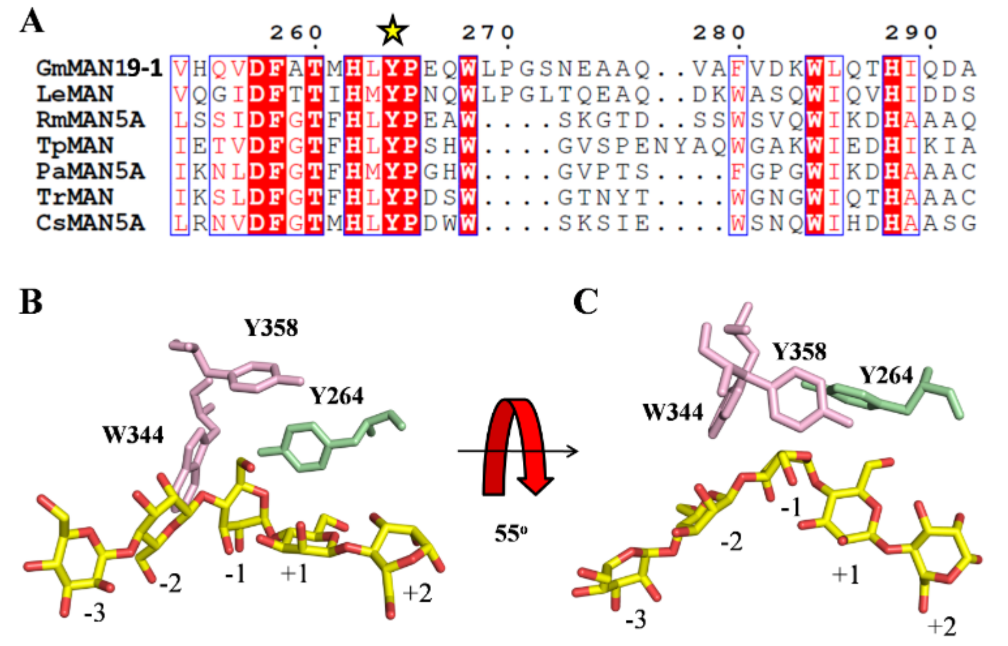

Figure S14. Y264 is highly conserved in different species and participate in the torsion of sugar at -1 subsite.

(A) Multiple sequence alignments of GmMAN19-1 with other similar β-mannanase structures based on structural comparison, the star points out the site of Y264. Y264 provide hydrophobic interaction to twist saccharide at -1 subsite, mannopentaose are drawn as sticks with carbon atoms in yellow. (B) front view ; (C) top view.

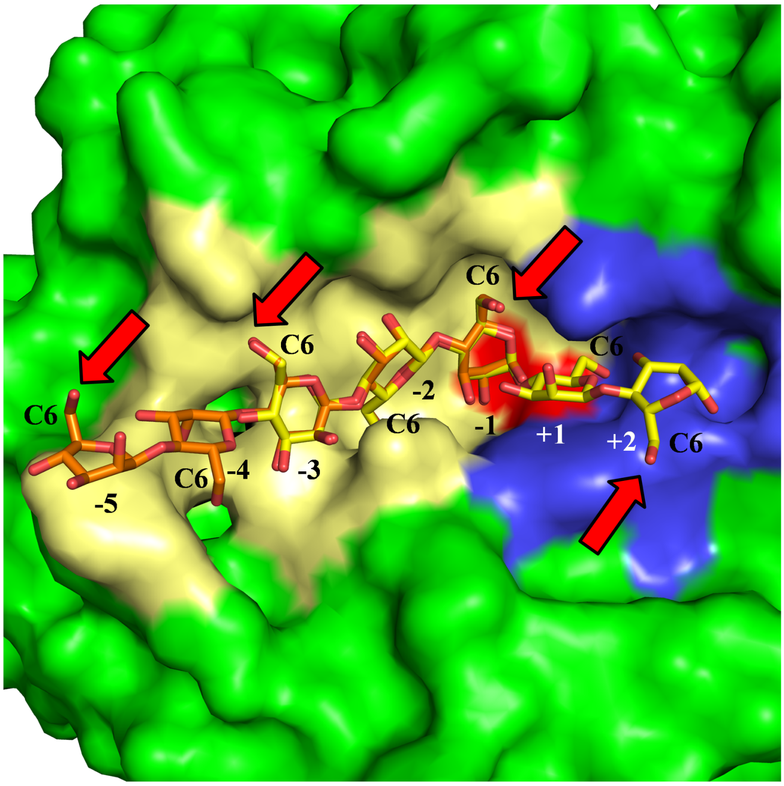

Figure S15. Accommodation of galactose side chain at surface binding site.

The graph highlights subsites +2, -1, -3, and -5 as potential positions for accommodating branch chains (indicated by red arrows). Mannopentaose is shown as sticks with carbon atoms colored in yellow and orange.

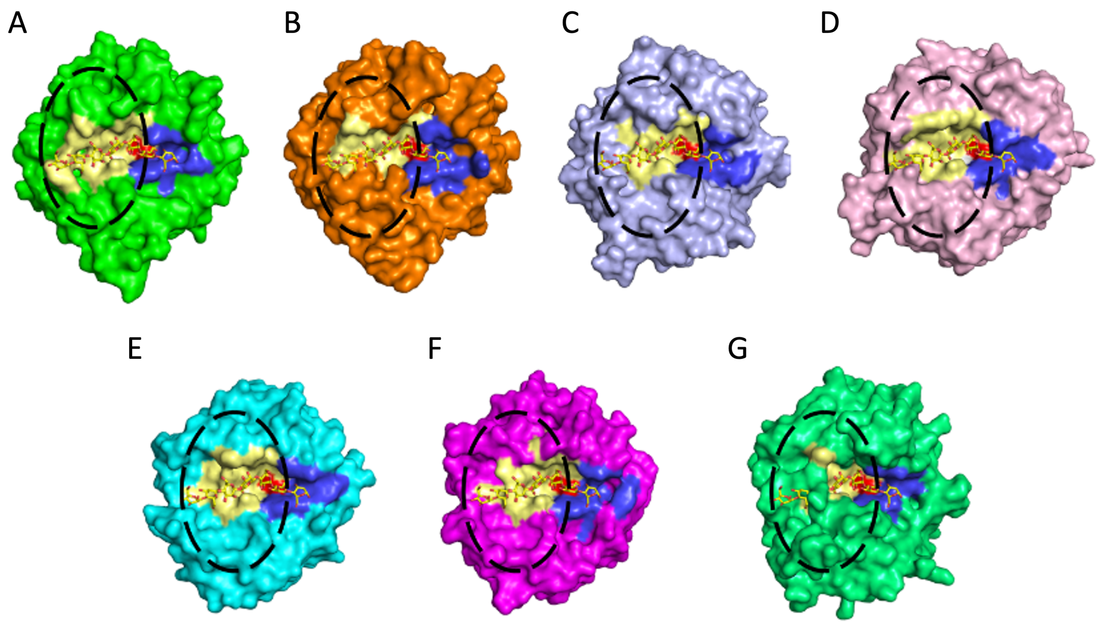

Figures S16. Difference of substrate binding clef between related six similar β-mannanases structures and GmMAN19-1.

Yellow area represents negative subsites, blue area represents positive subsites and two catalytic glutamates are colored red. All structures are shown as molecular surfaces. Black circles are used to point out the difference between the negative subsites. A, GmMAN19-1 (green)；B, LeMAN (1RH9, orange)；C, RmMAN5A (4QP0, blue)；D, TpMAN (6TNT, deep salmon)；E, PaMAN5A (3ZIZ, cyan)；F, TrMAN (1QNO, magentas)；G, CsMAN5A (4AWE, lime green), mannopentaose are drawn as sticks with carbon atoms in yellow.

References:

Chen, X., Cao, Y., Ding, Y., Lu, W., & Li, D. (2007). Cloning, functional expression and characterization of Aspergillus sulphureus beta-mannanase in Pichia pastoris. *J Biotechnol, 128*(3), 452-461. https://doi.org/10.1016/j.jbiotec.2006.11.003.

Huang, J. W., Chen, C. C., Huang, C. H., Huang, T. Y., Wu, T. H., Cheng, Y. S., . . . Guo, R. T. (2014). Improving the specific activity of beta-mannanase from Aspergillus niger BK01 by structure-based rational design. *Biochim Biophys Acta, 1844*(3), 663-669. https://doi.org/10.1016/j.bbapap.2014.01.011.

Li, J. F., Zhao, S. G., Tang, C. D., Wang, J. Q., & Wu, M. C. (2012). Cloning and functional expression of an acidophilic beta-mannanase gene (Anman5A) from Aspergillus niger LW-1 in Pichia pastoris. *J Agric Food Chem, 60*(3), 765-773. https://doi.org/10.1021/jf2041565.

Puchart, V., Vrsanska, M., Svoboda, P., Pohl, J., Ogel, Z. B., & Biely, P. (2004). Purification and characterization of two forms of endo-beta-1,4-mannanase from a thermotolerant fungus, Aspergillus fumigatus IMI 385708 (formerly Thermomyces lanuginosus IMI 158749). *Biochim Biophys Acta, 1674*(3), 239-250. https://doi.org/10.1016/j.bbagen.2004.06.022.

Wang, J., Zeng, D., Liu, G., Wang, S., & Yu, S. (2014). Truncation of a mannanase from Trichoderma harzianum improves its enzymatic properties and expression efficiency in Trichoderma reesei. *J Ind Microbiol Biotechnol, 41*(1), 125-133. https://doi.org/10.1007/s10295-013-1359-2.

Wang, Y., Azhar, S., Gandini, R., Divne, C., Ezcurra, I., & Aspeborg, H. (2015). Biochemical characterization of the novel endo-beta-mannanase AtMan5-2 from Arabidopsis thaliana. *Plant Sci, 241*, 151-163. https://doi.org/10.1016/j.plantsci.2015.10.002.

Wang, Y., Vilaplana, F., Brumer, H., & Aspeborg, H. (2014). Enzymatic characterization of a glycoside hydrolase family 5 subfamily 7 (GH5_7) mannanase from Arabidopsis thaliana. *Planta, 239*(3), 653-665. https://doi.org/10.1007/s00425-013-2005-y.
